# Performance of radiomics in the differential diagnosis of parotid tumors: a systematic review

**DOI:** 10.3389/fonc.2024.1383323

**Published:** 2024-07-25

**Authors:** Yilin Rao, Yuxi Ma, Jinghan Wang, Weiwei Xiao, Jiaqi Wu, Liang Shi, Ling Guo, Liyuan Fan

**Affiliations:** ^1^ Department of Prosthodontics, The Affiliated Stomatology Hospital, Southwest Medical University, Luzhou, Sichuan, China; ^2^ Luzhou Key Laboratory of Oral & Maxillofacial Reconstruction and Regeneration, The Affiliated Stomatological Hospital, Southwest Medical University, Luzhou, Sichuan, China

**Keywords:** meta-analysis, parotid tumors, radiomics, deep learning, machine learning

## Abstract

**Purpose:**

A systematic review and meta-analysis were conducted to evaluate the diagnostic precision of radiomics in the differential diagnosis of parotid tumors, considering the increasing utilization of radiomics in tumor diagnosis. Although some researchers have attempted to apply radiomics in this context, there is ongoing debate regarding its accuracy.

**Methods:**

Databases of PubMed, Cochrane, EMBASE, and Web of Science up to May 29, 2024 were systematically searched. The quality of included primary studies was assessed using the Radiomics Quality Score (RQS) checklist. The meta-analysis was performed utilizing a bivariate mixed-effects model.

**Results:**

A total of 39 primary studies were incorporated. The machine learning model relying on MRI radiomics for diagnosis malignant tumors of the parotid gland, demonstrated a sensitivity of 0.80 [95% CI: 0.74, 0.86], SROC of 0.89 [95% CI: 0.27-0.99] in the validation set. The machine learning model based on MRI radiomics for diagnosis malignant tumors of the parotid gland, exhibited a sensitivity of 0.83[95% CI: 0.76, 0.88], SROC of 0.89 [95% CI: 0.17-1.00] in the validation set. The models also demonstrated high predictive accuracy for benign lesions.

**Conclusion:**

There is great potential for radiomics-based models to improve the accuracy of diagnosing benign and malignant tumors of the parotid gland. To further enhance this potential, future studies should consider implementing standardized radiomics-based features, adopting more robust feature selection methods, and utilizing advanced model development tools. These measures can significantly improve the diagnostic accuracy of artificial intelligence algorithms in distinguishing between benign and malignant tumors of the parotid gland.

**Systematic review registration:**

https://www.crd.york.ac.uk/prospero/, identifier CRD42023434931.

## 1 Introduction

Salivary gland tumors represent 2.0-6.5% of head and neck tumors and account for 0.5% of all malignant tumors. Approximately 70% of salivary gland tumors are found in the parotid gland (1, 2). In accordance with the most recent WHO histological classification, salivary gland tumors encompass 22 malignant epithelial tumors and 14 benign epithelial tumors (3). The majority of parotid tumors, about 80%-85%, are benign, with pleomorphic adenomas (PA) being the most prevalent (accounting for approximately 65% of all parotid tumors), followed by Warthin tumors (constituting approximately 15%-20% of all parotid tumors) (2, 4). Malignant salivary gland tumors represent around 15%-30% of parotid tumors (2, 5).

Currently, the detection of parotid tumors primarily relies on fine needle aspiration cytology (FNA) using a small-caliber needle. This method is straightforward and minimally invasive. FNA is commonly used to differentiate between neoplastic and non-neoplastic lesions and to diagnose histological types of neoplastic lesions, such as pleomorphic adenoma and Warthin’s tumors in benign tumors (6, 7). It is also used to determine the malignancy level of malignant tumors. For benign cases, long-term follow-up or limited partial parotidectomy is usually sufficient. However, malignant parotid gland tumors require a more aggressive surgical approach (8). For example, patients with lower superficial malignancy may undergo conservative surgery to remove tumors while preserving the facial nerve, while those with higher malignancy may need total parotidectomy and neck dissection. Thus, a preoperative diagnosis of parotid tumors is crucial in determining the appropriate surgical approach (9).

However, due to sampling difficulties and tumor heterogeneity, fine needle aspiration cytology is sometimes inconclusive and may not accurately represent the true nature of the tumors. However, during the aspiration process, it will cause certain pain to the patient. Therefore, some researchers are currently conducting non-invasive screening studies, such as radiomics-based machine learning. However, there is still a lack of systematic evidence for its feasibility, which brings certain challenges for the advancement of radiomics-based machine learning in non-invasive screening for parotid tumors. Therefore, this systematic review was conducted. Furthermore, the risk of disseminating tumor cells, elevating the possibility of local recurrence, and occasionally, increasing the susceptibility to infections should also be taken into consideration (10). Therefore, a radiological assessment plays a crucial role in accurately determining the characteristics of the parotid gland (11). Currently, magnetic resonance imaging (MRI) and computed tomography (CT) are widely utilized for evaluating parotid tumors (12, 13). MRI provides precise evaluation of the invasion and boundaries of parotid tumors, making it a reliable method for assessing these tumors. Numerous studies have reported that MRI assists clinicians in making a differential diagnosis of parotid tumors (14). CT scans clearly demonstrate the contour and internal structure of the parotid gland, particularly after enhancement. They can accurately localize parotid gland masses, providing details such as the number, size, shape, boundaries, and infiltration into surrounding tissues (15). Nevertheless, these diagnostic techniques still have limitations, and there may be significant similarities in radiological features among different types of parotid tumors (16). Certain studies have indicated that alterations in the margins of parotid tumors may not necessarily imply malignancy, and heterogeneous enhancement characteristics cannot be reliably used to distinguish between benign parotid tumors and malignant tumors of the parotid gland (17, 18). Additionally, some benign parotid gland tumors may resemble malignant tumors due to the presence of cystic degeneration and necrotic areas (19). Furthermore, it carries the risk of spreading tumor cells, increasing the likelihood of local recurrence, and at times, raising the risk of infections.

The diagnostic accuracy of medical imaging can be limited by the subtle changes in features that may not be noticeable to the naked eye (20, 21). To overcome this limitation, Dutch researcher Lambin introduced the concept of radiomics in 2012, which aims to extract a large number of image features from radiation images using high throughput methods (22). Around the same time, Kumar proposed the idea of imaging omics, which involves extracting and analyzing quantitative image features from CT, PET, or MRI scans at high throughput (23). Radiomics, as a non-invasive and high-throughput post-processing technique, can provide a more comprehensive set of information than what can be discerned by the human eye alone. By converting numerous imaging features into high-dimensional mineable data, radiomics has made significant advancements in tumor diagnosis, treatment response assessment, and prognosis (24, 25). For patients with head and neck cancer, CT scans can not only predict HPV (P16) status in oropharyngeal squamous cell carcinoma (26), but also indicate hypoxic status (27), aiding in the differentiation of oropharyngeal carcinoma from hypopharyngeal carcinoma (28). Additionally, MRI radiologic features have been recognized as noninvasive, preoperative, and independent prognostic factors for head and neck squamous cell carcinoma (HNSCC) and nasopharyngeal carcinoma (NPC) in clinical practice (29, 30). Researchers have also attempted to incorporate radiomics into the early noninvasive differential diagnosis of benign and malignant tumors of the parotid gland. However, there is still a lack of systematic evidence regarding its differential value, which hinders the advancement of radiomics in this field. Therefore, this systematic review and meta-analysis were conducted to investigate the diagnostic accuracy of radiomics for benign and malignant tumors of the parotid gland.

## 2 Materials and methods

### 2.1 Study registration

This study was conducted according to the guidelines for systematic reviews and meta-analyses (PRISMA 2020) and was prospectively registered with PROSPERO (ID: CRD42023434931).

### 2.2 Eligibility criteria

#### 2.2.1 Inclusion criteria

(1) Patients with suspected malignant parotid tumors;
(2) A comprehensive machine learning model covering radiomics was constructed to detect the types of parotid gland lesions;
(3) A large number of studies may not have independent validation cohorts, but their contributions cannot be ignored, so such studies were also included in our systematic review;
(4) Studies reported in the English language.

#### 2.2.2 Exclusion criteria

(1) Study types: meta-analysis, reviews, guidelines, expert opinions, etc.;
(2) Studies solely conducted differential factor analysis and did not develop a complete machine learning model;
(3) Studies did not provide the following outcome indicators (ROC, c-statistic, c-index, sensitivity, specificity, accuracy, recovery rate, accuracy rate, confusion matrix, diagnostic fourfold table, F1 score, calibration curve);
(4) Studies with the number of cases less than 20; and
(5) Studies on segmentation of images without constructing complete machine learning models.

### 2.3 Data sources and search strategy

Databases of PubMed, Cochrane, Embase, and Web of Science up to May 5, 2023 were systematically searched. The search terms were designed by combining subject words and free words. The search was not limited by publication years or regions. In order to avoid the risk of missing newly published literature, a supplementary search of each database was conducted on May 29, 2024. The complete search strategy is detailed in **Supplementary Material, Annex 1**.

### 2.4 Study selection and data extraction

The literature that was obtained was imported into Endnote for the purpose of automatically and manually removing any duplicate publications. Following this, the titles and abstracts were carefully assessed to exclude studies that did not meet the specified inclusion criteria. Lastly, the full texts of the initially eligible studies were downloaded and thoroughly examined to identify primary studies that met the requirements of this systematic review.

Before performing data extraction, a standardized data extraction spreadsheet was created. This spreadsheet included the following information: title, first author, year of publication, country, study type, patient source, radiomics source, complete image protocol, acquisition order, number of investigators involved, whether repeated measurement experiments were conducted at different times with different image parameters, image region of interest (ROI) region, segmentation software, texture extraction software, diagnostic events, all diagnostic events, number of cases, all cases, number of training sets, diagnostic events, number of training sets, number of case validation sets generated, method validation set, number of diagnostic events, number of case validation sets, variable screening method, modeling variables using model type, whether to establish radiomics scores, overfit assessment, whether to expose codes and data models, and assessment metrics.

The literature screening, data extraction, and data cross-verification were conducted by two investigators independently. In cases of disagreement, a third investigator was involved in discussions and decision-making.

### 2.5 Assessment of study quality

Two investigators assessed the methodological quality of the included studies and the risk of bias using Radiomics Quality Scores (RQS) (31) (**Supplementary Material, Annex 2**) and cross-checked upon completion. In case of disputes, a third investigator was consulted to aid in the adjudication.

### 2.6 Outcomes

Our systematic review focused on two primary outcomes: the c-index, which measures the overall precision of the model, and the sensitivity and specificity, which assess the accuracy of parotid tumor prediction. Additionally, it is noted that certain studies incorporated clinical indications when developing radiomics models. Therefore, our secondary outcomes involved analyzing the frequency of clinical indication variables utilized in machine learning models.

### 2.7 Synthesis methods

A meta-analysis was performed to evaluate the overall accuracy of the machine learning models by assessing the c-index. In certain primary studies, the 95% confidence interval and standard error for the c-index were not available. To address this, the approach outlined by Debray TP et al. (32) was followed to estimate the standard error of the c-index. Considering variations in the inclusion variables and inconsistent parameters across different machine learning models, preference was given to the utilization of random-effects models in our meta-analysis of the c-index.

Furthermore, conducted meta-analyses were conducted to evaluate the sensitivity and specificity. These analyses were performed using binary mixed-effects models. In most of the primary studies, the diagnostic fourfold table was not provided. In these cases, two methods were employed to calculate the diagnostic fourfold table:

1. Sensitivity, specificity, and accuracy (precision) were combined with the number of case; 2. Sensitivity and specificity were extracted utilizing the best Youden index and combined with the number of cases. The meta-analyses in this study were conducted using R 4.2.0 (R Development Core Team, Vienna, www.R-project.org).

## 3 Results

### 3.1 Study selection

**Figure 1:**
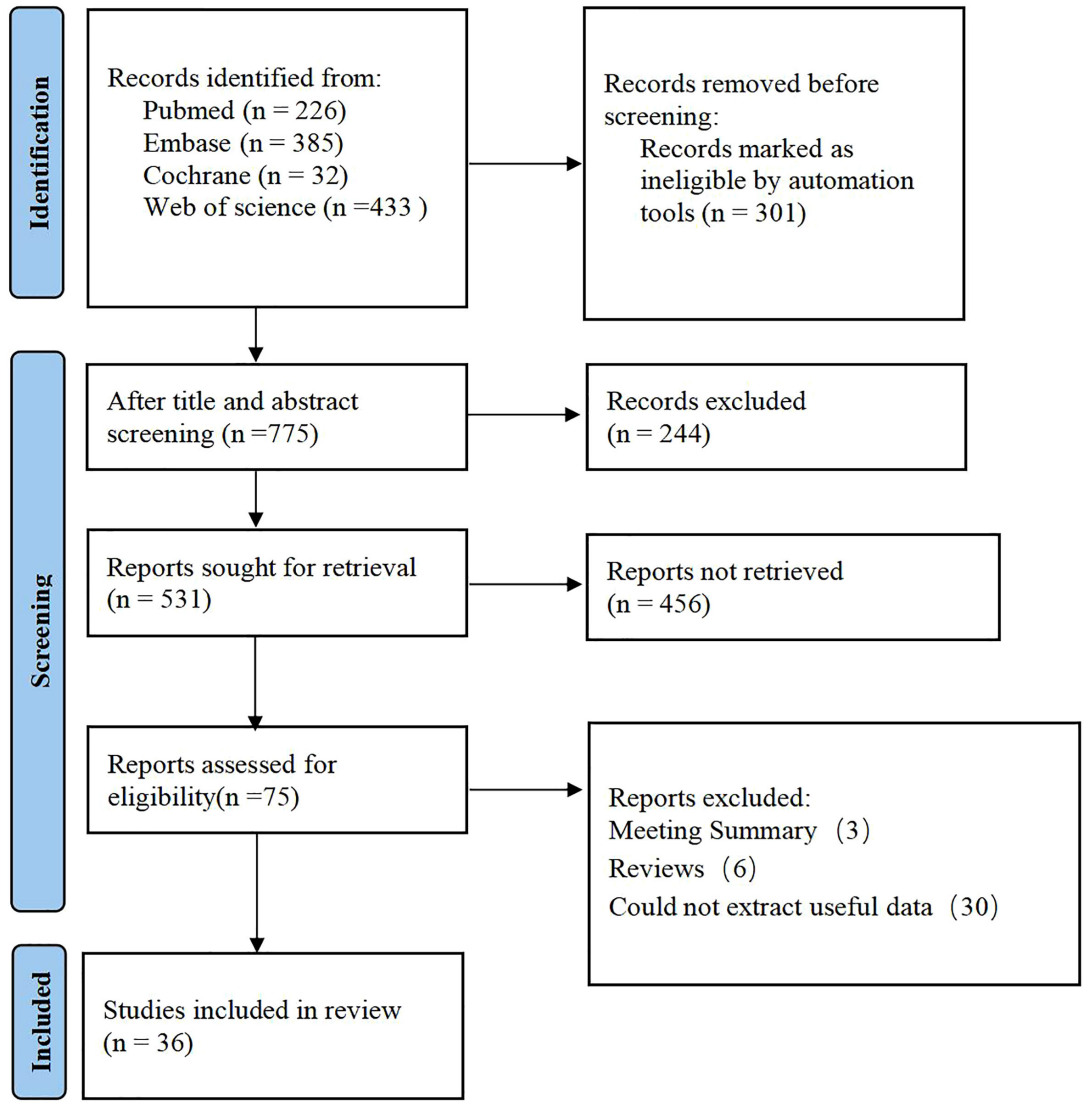
Literature screening process.

A total of 1076 articles were retrieved from the database, among which 301 duplicate publications were automatically identified through software marking, and 244 publications were manually identified. After reviewing the titles and abstracts, 75 primary studies that were initially eligible were retained. Upon downloading and reading the full texts, 3 summary, 6 reviews and 30 studies with incomplete data were excluded. Eventually, 36 primary studies were included (33–68) (**Figure 1**).

### 3.2 Study characteristics

The 36 studies included in this analysis were mostly published between 2020 and 2024. They were conducted in various countries including China, Italy, Japan, and Canada. Among these studies, 24 specifically focused on the differential diagnosis of malignant parotid tumors (33–37, 40, 41, 43, 44, 47, 48, 50, 52–57, 59–61, 63, 66, 67). There were 12 studies on the differential diagnosis of benign parotid tumors (39, 42, 45, 46, 48, 50, 51, 58, 62, 64, 65, 68), 9 studies on the differential diagnosis of pleomorphic adenoma (42, 46, 48, 51, 58, 62, 64, 65, 68), and 3 studies on the differential diagnosis of Warthin’s tumors (38, 39, 45). In these 36 studies, MRI radiomics was utilized in 19 studies (33, 36, 37, 39–41, 43, 45, 48, 50, 52–55, 57, 60, 62, 64, 66), while CT radiomics was used in 16 studies (34, 35, 38, 42, 44, 46–48, 51, 56, 59, 61, 63, 65, 67, 68). One study employed both MRI and CT radiomics (58). Among the 19 studies that used radiomic features as modeling variables (35–39, 43, 44, 47, 50, 55, 59–63, 65–68), 12 studies incorporated clinical features and radiomic features, or radiomic features alone, into their modeling variables (34, 42, 45, 46, 49, 51–54, 56, 58, 64). Two studies (40, 41) considered ADC values as a single modeling variable for the differential diagnosis of parotid lesions, while three studies (33, 48, 57) included them as one of the modeling variables.

### 3.3 Assessment of study quality

The included studies failed to consider the variations in output images produced by the same equipment under different parameters. They also did not conduct multiple measurements at different times in the same individual, perform prospective registration, explore the detection further and discuss biological correlation, demonstrate the level of agreement with the “gold standard,” or construct clinical impact curves. Consequently, they received a score of zero on the Radiomics Quality Score (RQS). Out of the 33 studies, random sampling was predominantly used for internal validation. Among them, 6 studies had independent external validation (35, 45, 49, 54, 55, 61). Eight studies provided an analysis of cut-off values (45, 46, 48, 49, 55, 58, 61, 64, 66), while 8 studies constructed calibration curves (45, 46, 48, 49, 54, 56, 61, 64). The average RQS score for the included studies was 6.83 (**Supplementary Material, Annex 3**).

### 3.4 Meta-analysis

#### 3.4.1 Diagnosis of malignant tumors of parotid gland

##### 3.4.1.1 CT radiomics

**Figure 2:**
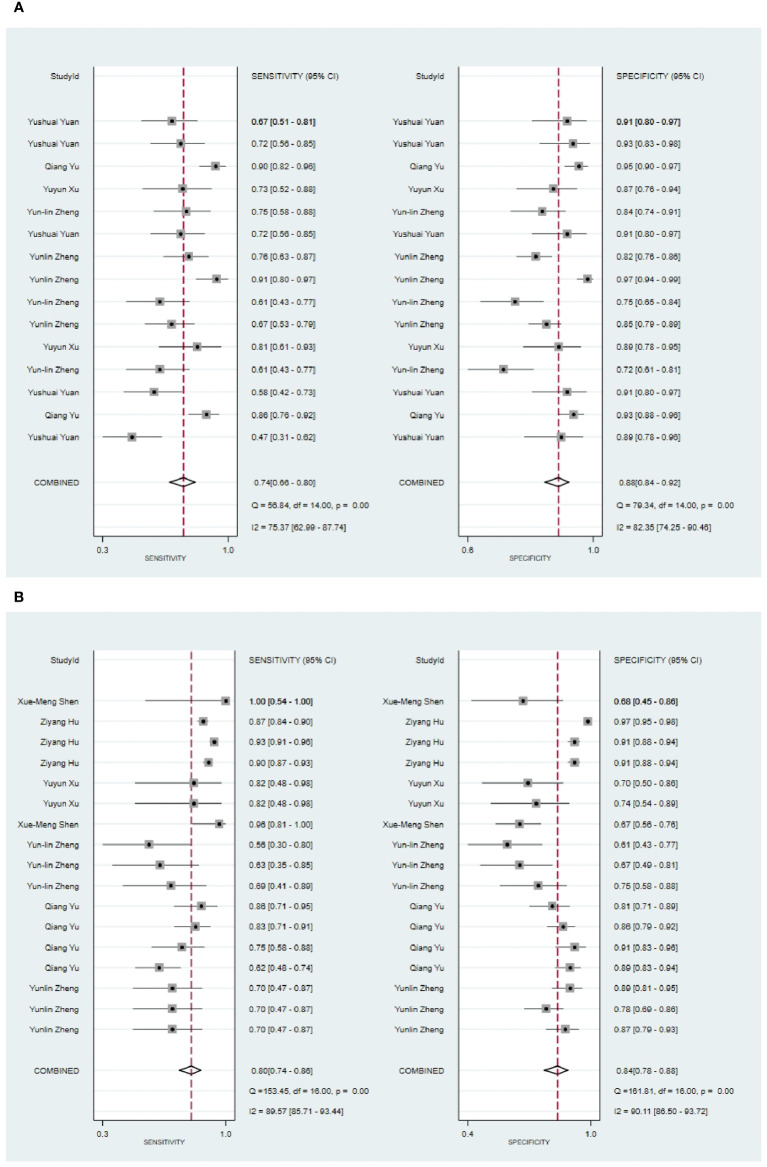
The Forest plot of radiomics based on CT for the diagnosis of malignant tumors. **(A)** The training set; **(B)** The validation set.

Five eligible studies reported CT-based models for the diagnosis of malignant tumors of parotid gland (34, 35, 42, 47, 61). CT-based models in the training set demonstrated the following performance metrics: a sensitivity of 0.74 [95% CI: 0.66, 0.80], a specificity of 0.88 [95% CI: 0.84, 0.92], a PLR of 6.4 [95% CI: 4.4, 9.3], an NLR of 0.30 [95% CI: 0.23, 0.40], a DOR of 21 [95% CI: 11, 40], and an SROC curve value of 0.89 [95% CI: 1.00-0.00] (**Figure 2A**; **Supplementary Figure S1**). Deek’s funnel plot reveals the presence of publication bias (P) among the models. The prevalence of malignant parotid tumors estimated in the included studies serves as the prior probability of the diagnostic experiment. It should be noted that the actual probability of a malignant parotid tumor was 73% if the model identified it as such (**Supplementary Figures S2**, **S3**).

In the validation set (34, 35, 42, 44, 61, 63, 67), CT-based models demonstrated a sensitivity of 0.80 [95% CI: 0.74, 0.86], a specificity of 0.84 [95% CI: 0.78, 0.88], a PLR of 4.9 [95% CI: 3.5, 7.0], an NLR of 0.23[95% CI: 0.17, 0.33], a DOR of 22 [95% CI: 11, 39], and an SROC of 0.89 [95% CI: 0.27-0.99] (**Figure 2B**; **Supplementary Figure S4**). Deek’s funnel plot revealed no evidence of publication bias among the models (P). Taking into account the estimated prevalence of malignant parotid tumors from the studies included as the prior probability for the diagnostic experiment, the observed probability of a malignant parotid tumor was 79% if the model detected a malignant parotid tumor (**Supplementary Figures S5**, **S6**).

##### 3.4.1.2 MRI radiomics

**Figure 3:**
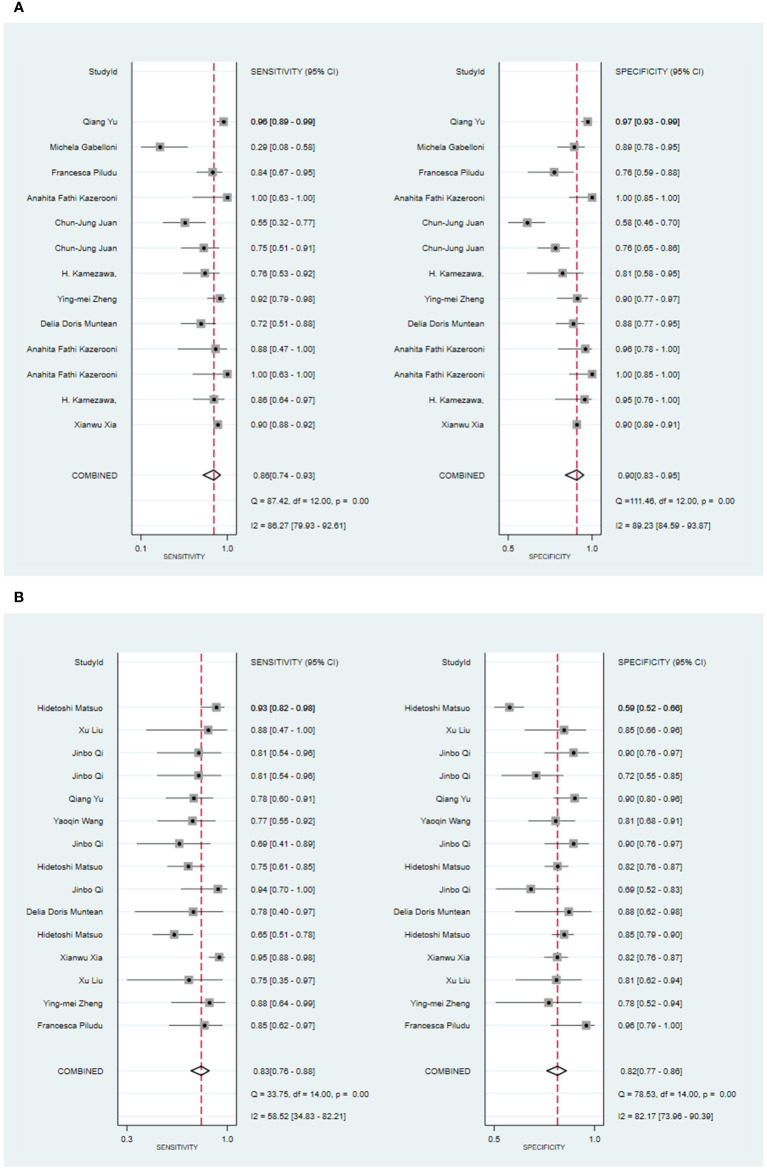
The Forest plot of radiomics based on MRI for the diagnosis of malignant tumors. **(A)** The training set; **(B)** The validation set.

Seven primary studies reported MRI-based models for the diagnosis of malignant tumors of the parotid gland (36, 43, 50, 54–57, 66). MRI-based models demonstrated a sensitivity of 0.86 [95% CI: 0.74, 0.93], a specificity of 0.90 [95% CI: 0.83, 0.95], a PLR of 9.2 [95% CI: 4.5, 19.0], an NLR of 0.14 [95% CI: 0.07, 0.31], a PLR of 8.8 [95% CI: 4.6, 16.9], an NLR of 0.16 [95% CI: 0.08, 0.31], a DOR of 56 [95% CI: 16, 194], and an SROC of 0.94 [95% CI: 0.92 - 0.96] (**Figure 3A**; **Supplementary Figure S7**). Deek’s funnel plot illustrated that there was no evidence of publication bias among the models analyzed (P). By considering the prevalence of malignant parotid tumors estimated in the included studies as the prior probability for the diagnostic experiment, it was found that the actual probability of a malignant parotid tumor was 78% if the model identified it as such (**Supplementary Figures S8**, **S9**).

In the validation set, nine primary studies have reported models based on MRI for the diagnosis of malignant tumors in the parotid gland (33, 37, 43, 48, 54–56, 61, 66). MRI-based models demonstrated a sensitivity of 0.83 [95% CI: 0.76, 0.88], a specificity of 0.82 [95% CI: 0.77, 0.86], a PLR of 4.6 [95% CI: 3.6, 5.9], an NLR of 0.21 [95% CI: 0.15, 0.29], a DOR of 22 [95% CI: 15, 30], and an SROC of 0.89 [95% CI: 0.17-1.00] (**Figure 3B**; **Supplementary Figure S10**). Deek’s funnel plot did not indicate any signs of publication bias among the models (P). Taking into account the prevalence of pleomorphic adenoma estimated in the studies considered as the prior probability for the diagnostic experiment, the actual probability of a parotid tumor being malignant was 64% if the model identified it as such (**Supplementary Figures S11**, **S12**).

##### 3.4.1.3 Clinical features

**Figure 4:**
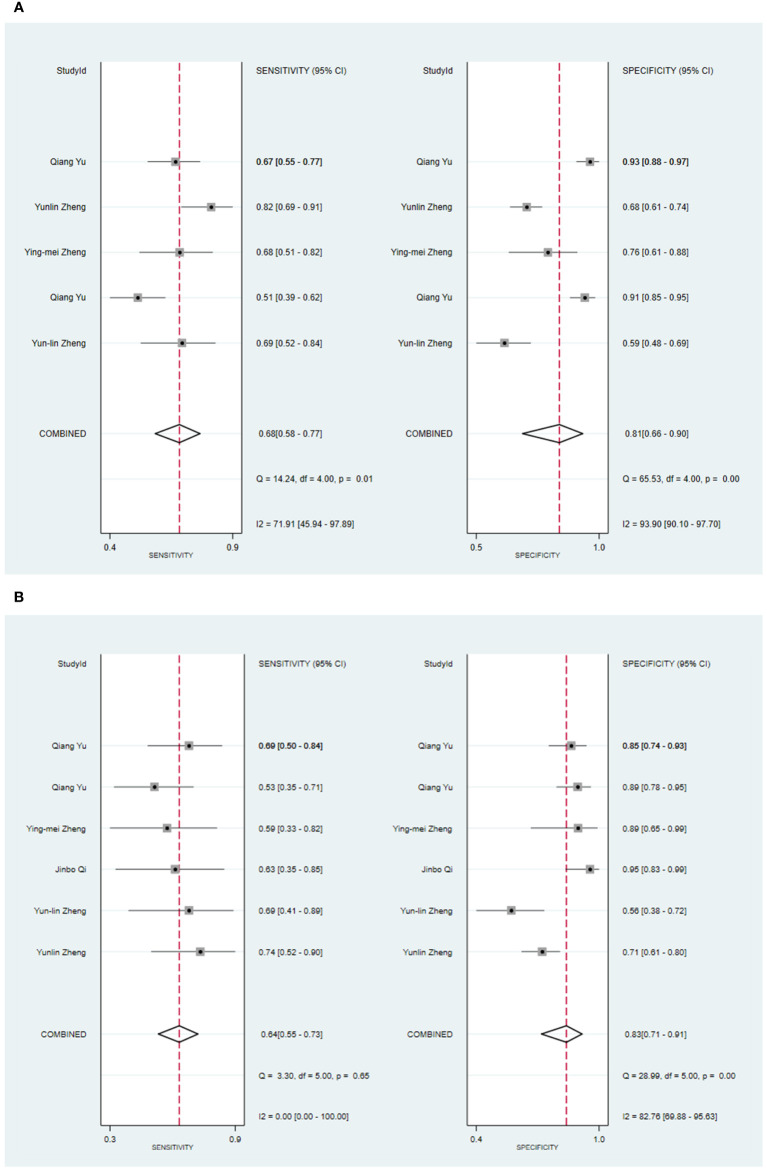
The Forest plot based on clinical features for the diagnosis of malignant tumors. **(A)** The training set; **(B)** The validation set.

Four primary studies have presented models based on clinical features to diagnose malignant tumors of the parotid gland (34, 42, 54, 56). The machine learning model, which solely relies on clinical features, exhibited a sensitivity of 0.68 [95% CI: 0.58, 0.77], a specificity of 0.81 [95% CI: 0.66, 0.90], a PLR of 3.6 [95% CI: 2.1, 6.1], an NLR of 0.39 [95% CI: 0.31, 0.50], a DOR of 9 [95% CI: 5, 16], and an SROC of 0.78 [95% CI: 1.00-0.00] (**Figure 4A**; **Supplementary Figure S13**). Deek’s funnel plot did not indicate any publication bias across the different models (P). Considering the prevalence of malignant parotid tumors estimated in the included studies as the prior probability of the diagnostic experiment, the actual probability of a malignant parotid tumor reached 60% if the model identified it (**Supplementary Figures S14**, **S15**). In the validation set, five studies described models based on clinical features for diagnosing malignant tumors of the parotid gland (34, 42, 48, 54, 56). The machine learning model, relying on clinical features alone, demonstrated a sensitivity of 0.64 [95% CI: 0.55, 0.73], a specificity of 0.83 [95% CI: 0.71, 0.91], a PLR of 3.8 [95% CI: 2.2, 6.6], an NLR of 0.43 [95% CI: 0.34, 0.54], a DOR of 9 [95% CI: 5, 17], and an SROC of 0.73 [95% CI: 0.19-0.97] (**Figure 4B**; **Supplementary Figure S16**). Deek’s funnel plot indicated the presence of publication bias among the models (P). With the prevalence of malignant parotid tumors estimated in the included studies as the prior probability of the diagnostic experiment, the actual probability of a malignant parotid tumor reached 62% when the model identified it (**Supplementary Figures S17**, **S18**).

##### 3.4.1.4 Integration of radiomic features with clinical features

**Figure 5:**
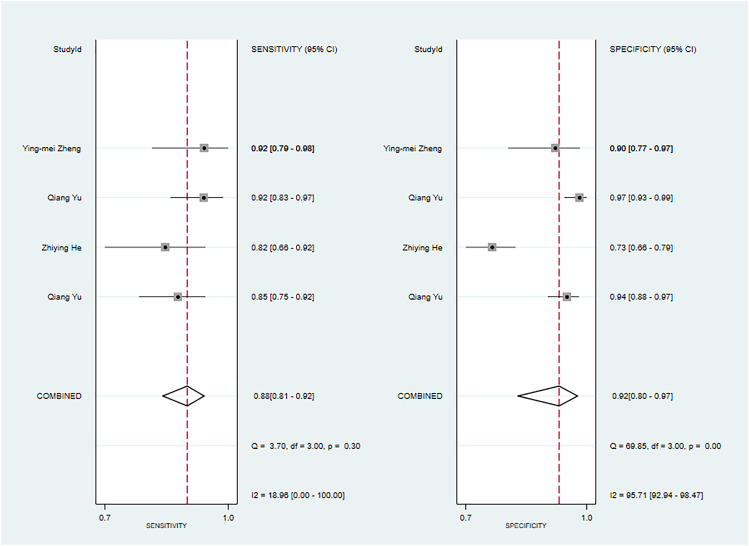
The Forest plot based on MRI-based models combined with clinical features for the diagnosis of malignant tumors (The training set).

**Figure 6:**
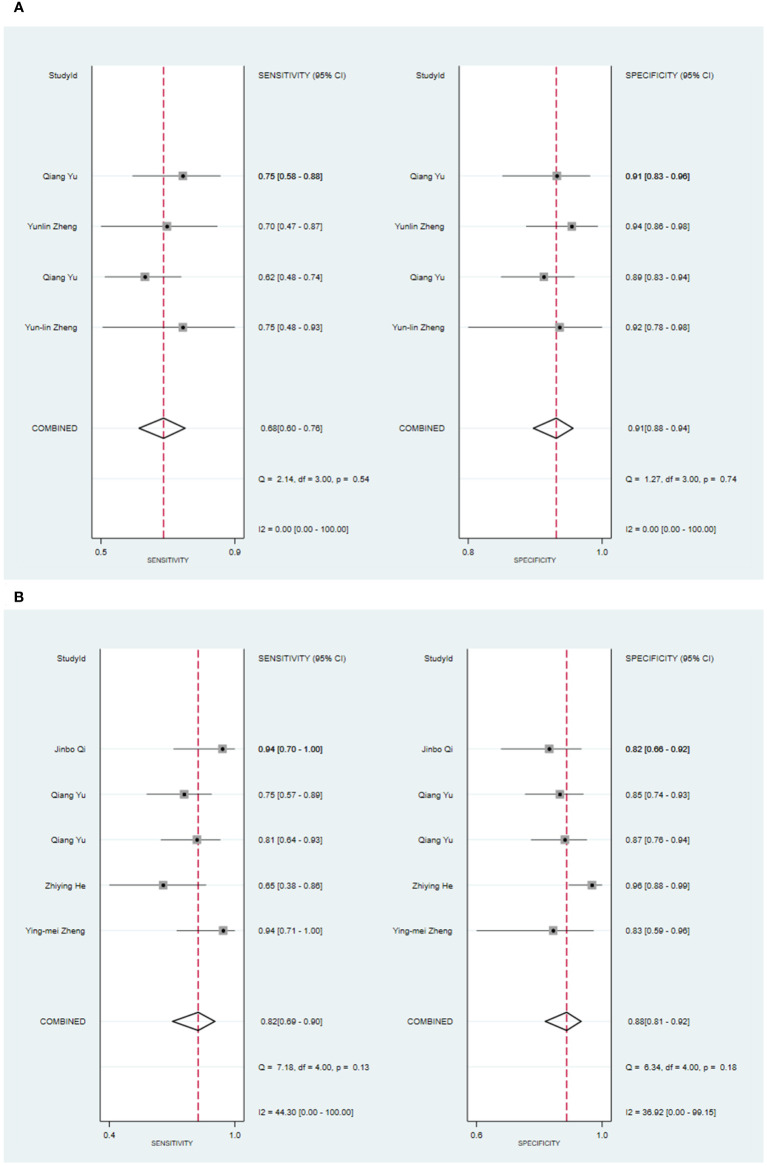
The Forest plot based on CT-based models combined with clinical features for the diagnosis of malignant tumors. **(A)** The training set; **(B)** The validation set.

Three primary studies have reported models that models based on MRI combined with clinical features for the diagnosis of malignant tumors of the parotid gland (52, 54, 56). The machine learning model, which incorporated radiomic features in addition to clinical attributes, demonstrated a sensitivity of 0.88 [95% CI: 0.81, 0.92], a specificity of 0.92 [95% CI: 0.80, 0.97], a PLR of 10.4 [95% CI: 4.1, 26.6], an NLR of 0.13 [95% CI: 0.08, 0.22], a DOR of 78 [95% CI: 21, 289], and an SROC of 0.93 [95% CI: 1.00-0.00] (**Figure 5**; **Supplementary Figure S19**). Deek’s funnel plot analysis revealed no evidence of publication bias between the models (P). By using the prevalence of malignant parotid tumors estimated from the included studies as the prior probability of the diagnostic experiment, the actual probability of identifying a malignant parotid tumor using the model was 91% (**Supplementary Figures S20**, **S21**). In the validation set, there were 3 primary studies that presented models using CT and clinical features for the diagnosis of malignant tumors of the parotid gland [34.35,42]. These machine learning models demonstrated a sensitivity of 0.68[95% CI: 0.60, 0.76], a specificity of 0.91[95% CI: 0.88, 0.94], a PLR of 7.7[95% CI: 5.4, 11.1], an NLR of 0.35[95% CI: 0.27, 0.45], a DOR of 22[95% CI: 13, 38], and an SROC of 0.89[95% CI: 1.00 - 0.00] (**Figure 6A**; **Supplementary Figure S22**). The funnel plot devised by Deek indicated the presence of publication bias among the models (P). Considering the prevalence of malignant parotid tumors estimated in the included studies as the prior probability of the diagnostic experiment, the actual probability of having a malignant parotid tumor was 75% if the model identified the tumor as malignant (**Supplementary Figures S23**, **S24**). Five primary studies have presented MRI-based models combined with clinical features for diagnosing malignant tumors of the parotid gland (48, 52, 54, 56). By utilizing radiomic features together with clinical features, the machine learning model exhibited a sensitivity of 0.82 [95% CI: 0.69, 0.90], a specificity of 0.88 [95% CI: 0.81, 0.92], a PLR of 6.6 [95% CI: 4.4, 10.1], an NLR of 0.21 [95% CI: 0.12, 0.35], a DOR of 32 [95% CI: 17, 56], and an SROC of 0.92 [95% CI: 1.00-0.00] (**Figure 6B**; **Supplementary Figure S25**). The Deek’s funnel plot indicated the presence of publication bias among the models (P). When considering the prevalence of malignant parotid tumors estimated in the included studies as the prior probability of the diagnostic experiment, the actual probability of a malignant parotid tumor was determined to be 75% if the model identified it as malignant (**Supplementary Figures S26**, **S27**).

One study found that for the diagnosis of Warthin’s tumors, models utilizing either clinical features alone or radiomic features (MRI) combined with clinical features were reported (45). In the training set, the machine learning model solely based on clinical features demonstrated a sensitivity of 0.6829 and a specificity of 0.8235. On the other hand, the LR model, which incorporated radiomic features (MRI) along with clinical features, showed a higher sensitivity of 0.9268 and a specificity of 0.8529. When validated, the model exhibited a sensitivity of 0.8571 and a specificity of 0.625. Another study focused on the diagnosis of Warthin’s tumors using ADC values exclusively (42). The machine learning model constructed based on ADC values achieved a sensitivity of 0.83 and a specificity of 0.8. In the realm of pleomorphic adenoma diagnosis, one study reported models combining radiomic features (MRI) with clinical features (64). In the training set, the LR model incorporating radiomic features (MRI) demonstrated a sensitivity of 0.875 and a specificity of 0.9524. Lastly, two primary studies reported models for the diagnosis of malignant tumors of the parotid gland using radiomic features (CT) combined with clinical features (34, 42). In the training set, the SVM model relying on these combined features achieved a sensitivity of 0.763~0.797 and a specificity of 0.912~0.953. When validated, this SVM model demonstrated a sensitivity of 0.696~0.755 and a specificity of 0.904~0.935.

##### 3.4.1.5 ADC

**Figure 7:**
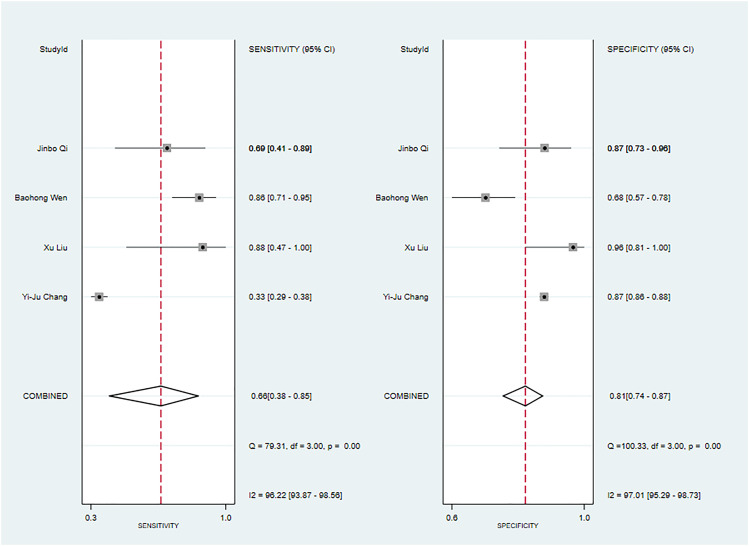
The Forest plot based on ADC values alone for the diagnosis of malignant tumors (The validation set).

Two primary studies have reported models solely based on ADC values for diagnosing malignant tumors of the parotid gland (39, 57). In the training set, the machine learning model relying solely on ADC values demonstrated a sensitivity of [0.24542~0.87583] and a specificity of [0.18997~0.80317]. Similarly, in the validation set, four primary studies reported models solely based on ADC values for diagnosing malignant tumors of the parotid gland (33, 39, 41, 48). The machine learning model, utilizing only ADC values, exhibited a sensitivity of 0.66 [95% CI: 0.38, 0.85], a specificity of 0.81 [95% CI: 0.74, 0.87], a PLR of 3.4 [95% CI: 2.7, 4.4], an NLR of 0.43 [95% CI: 0.22, 0.83], a DOR of 8 [95% CI: 3, 19], and an SROC of 0.83 [95% CI: 1.00-0.00] (**Figure 7**; **Supplementary Figure S28**). It was indicated by Deek’s funnel plot that publication bias was present among the models (P). Considering the estimated prevalence of malignant tumors in the parotid gland from the primary studies in this systematic review as the prior probability, the nomogram illustrated that the model could correctly identify malignant tumors of the parotid gland with a probability of **43%** (**Supplementary Figures S29**, **S30**).

#### 3.4.2 Diagnosis of benign tumors of parotid gland

##### 3.4.2.1 Diagnosis of Warthin’s tumors

###### 3.4.2.1.1 Ct Radiomics

**Figure 8:**
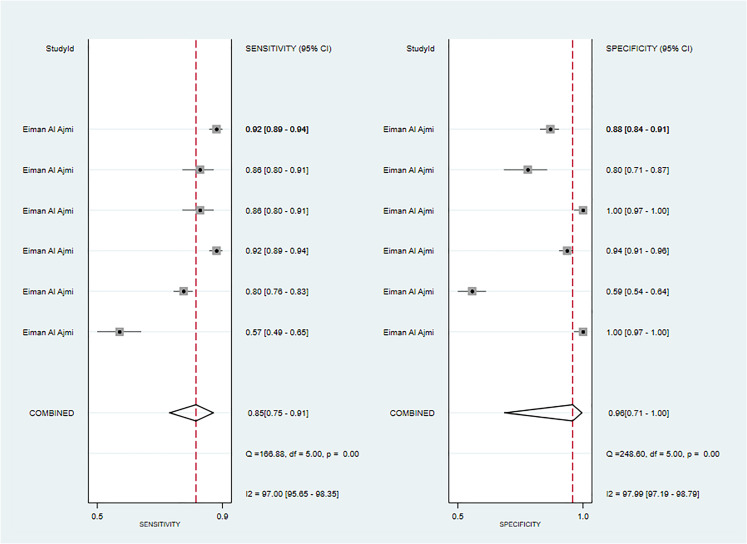
The Forest plot of radiomics based on CT-based for the diagnosis of Warthin’s tumors (The validation set).

One primary study reported CT-based models for the diagnosis of Warthin’s tumors (38). In the training set, the CT-based models constructed by RF had a sensitivity of [0.89~0.94] and a specificity of [0.67~1], respectively. In the validation set, six CT-based models (10) constructed by RF had a sensitivity of 0.85[95%CI:0.75,0.91] and a specificity of 0.96[95%CI:0.71,1.00], a PLR of 22.0[95%CI:2.5,197.7], an NLR of 0.16[95%CI:0.10,0.26], and a DOR of 137[95%CI:16,1155], and an SROC of 0.92[95%CI:0.90-0.94] (**Figure 8**; **Supplementary Figure S30**). Deek's funnel plot revealed no evidence of publication bias between models (P). With the prevalence of Warthin's tumors estimated in the included studies as the prior probability of the diagnostic experiment, the actual probability of Warthin’s tumors was 97% if the model identified a Warthin’s tumor (**Supplementary Figures S32**, **S33**).

###### 3.4.2.1.2 MRI Radiomics

**Figure 9:**
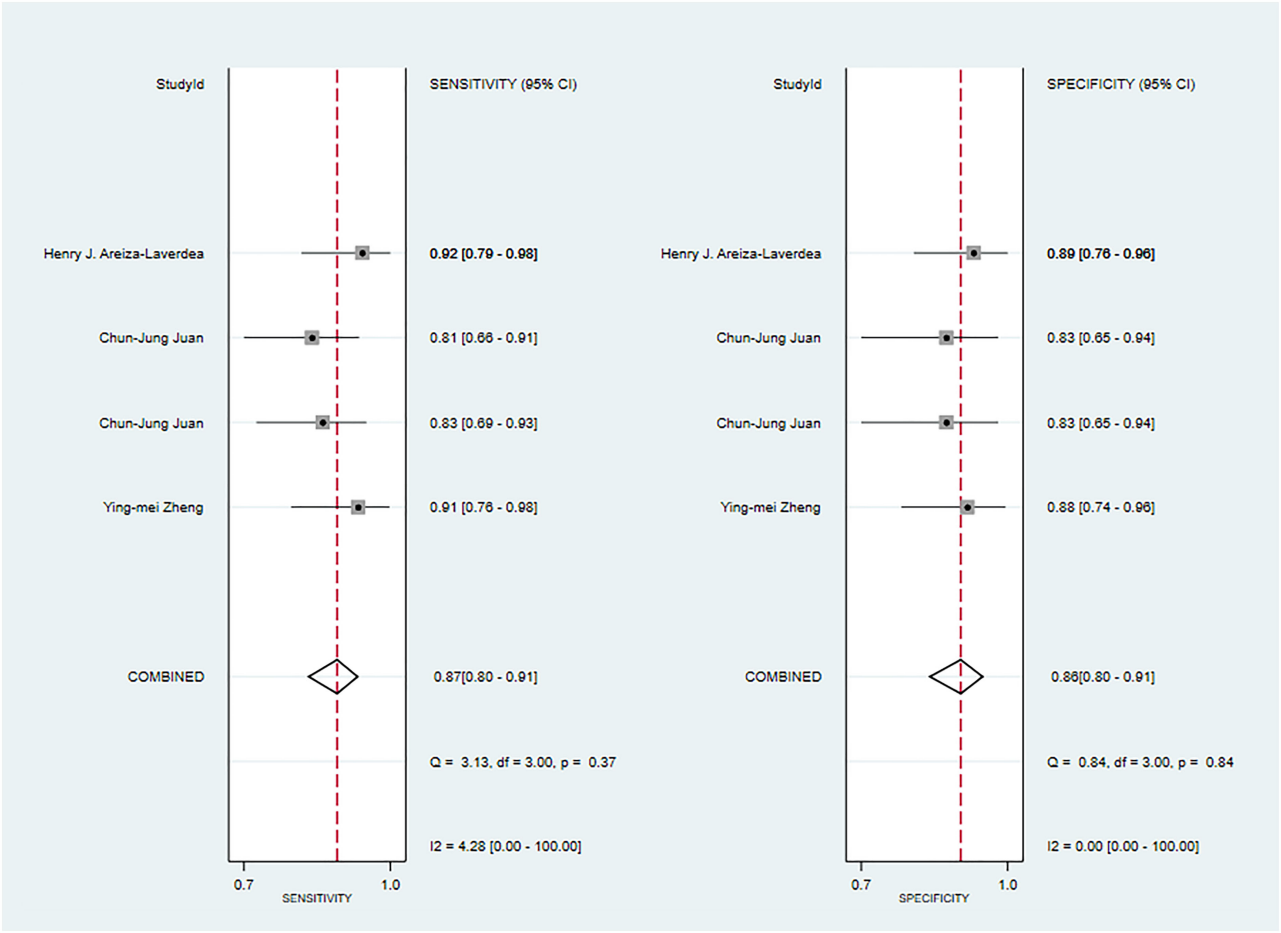
The Forest plot of radiomics based on MRI-based for the diagnosis of Warthin’s tumors (The training set).

Three primary studies reported MRI-based models for the diagnosis of Warthin's tumors (39, 45, 50), and MRI-based models demonstrated a sensitivity of 0.87[95%CI:0.80,0.91], a specificity of 0.86[95%CI:0.80,0.91], a PLR of 6.4[95%CI:4.2,9.6], an NLR of 0.16[95%CI:0.10,0.23], a DOR of 41[95%CI:21,79], and a SROC of 0.93[95%CI:0.90-0.95] (**Figure 9**; **Supplementary Figure S34**). Deek's funnel plot indicated the absence of publication bias between models (P). With the prevalence of Warthin's tumors estimated in the included studies as the prior probability of the diagnostic experiment, the actual probability of Warthin’s tumors was 87% if the model identified a Warthin’s tumor (**Supplementary Figures S35**, **S36**). In the validation set, one study (23) constructed an LR model, which had a sensitivity of 0.78 and a specificity of 0.87.

##### 3.4.2.2 Diagnosis of pleomorphic adenoma

###### 3.4.2.2.1 Ct Radiomics

**Figure 10:**
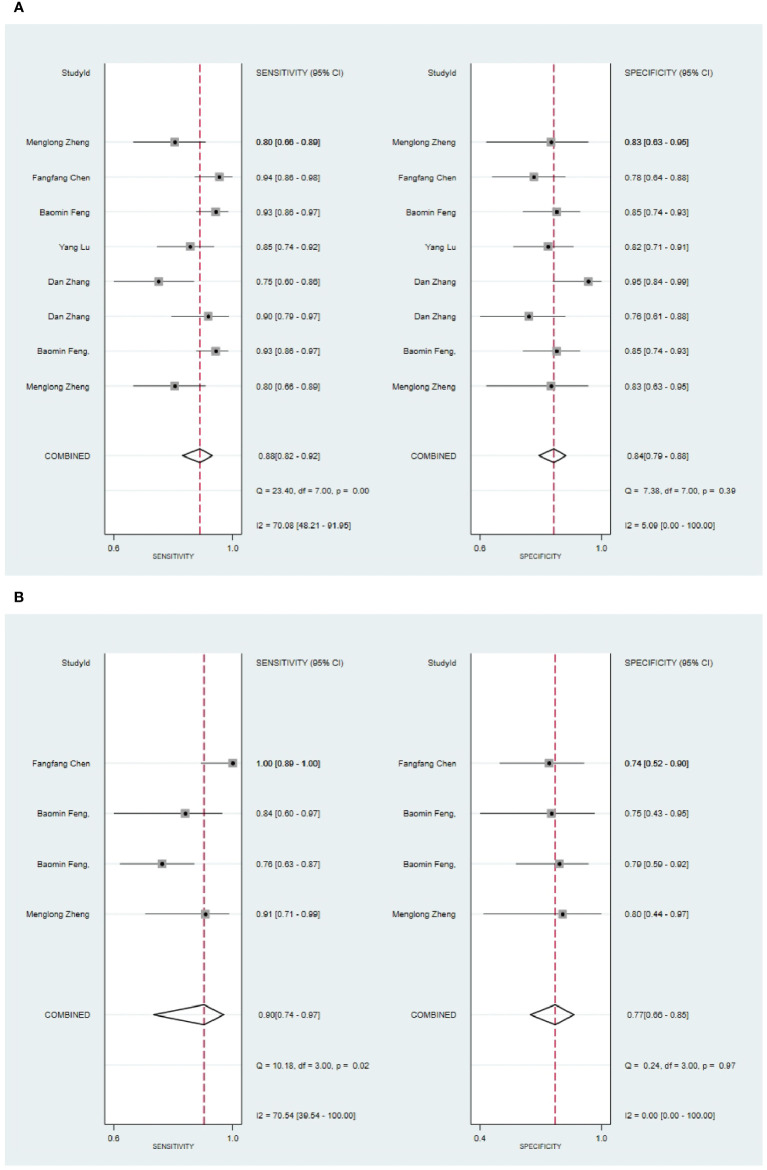
The Forest plot of radiomics based on CT-based for the diagnosis of pleomorphic adenoma. **(A)** The training set; **(B)** The validation set.

Five studies reported CT-based models for the diagnosis of pleomorphic adenoma (46, 49, 51, 65, 68). In the training set, CT-based models had a sensitivity of 0.87[95%CI:0.81,0.92], a specificity of 0.84[95%CI:0.78,0.89], a PLR of 5.5[95%CI:4.0,7.6], an NLR of 0.15[95%CI: 0.10, 0.23], a DOR of 37[95%CI:23, 59], and a SROC of 0.92 [95%CI:0.89-0.94] (**Figure 10A**; **Supplementary Figure S37**). Deek’s funnel plot indicated no evidence of publication bias between models (P). With the prevalence of pleomorphic adenoma estimated in the included studies as the prior probability of the diagnostic experiment, the actual probability of pleomorphic adenoma was 89% if the model identified a pleomorphic adenoma (**Supplementary Figures S38**, **S39**). In the validation set, six studies reported CT-based models for the diagnosis of pleomorphic adenoma, CT-based models had a sensitivity of 0.90[95%CI:0.74,0.97], a specificity of 0.77[95%CI:0.66,0.85], a PLR of 3.9[95%CI:2.5,5.9], an NLR of 0.12[95%CI: 0.04, 0.37], a DOR of 31[95%CI: 9,112], and a SROC of 0.80[95%CI:0.76- 0.83] (**Figure 10B**; **Supplementary Figure S40**). Deek’s funnel plot indicated no evidence of publication bias between models (P). With the prevalence of pleomorphic adenoma estimated in the included studies as the prior probability of the diagnostic experiment, the actual probability of pleomorphic adenoma was 87% if the model identified a pleomorphic adenoma (**Supplementary Figures S41, S42**) (46, 49, 69).

###### 3.4.2.2.2 MRI Radiomics

**Figure 11:**
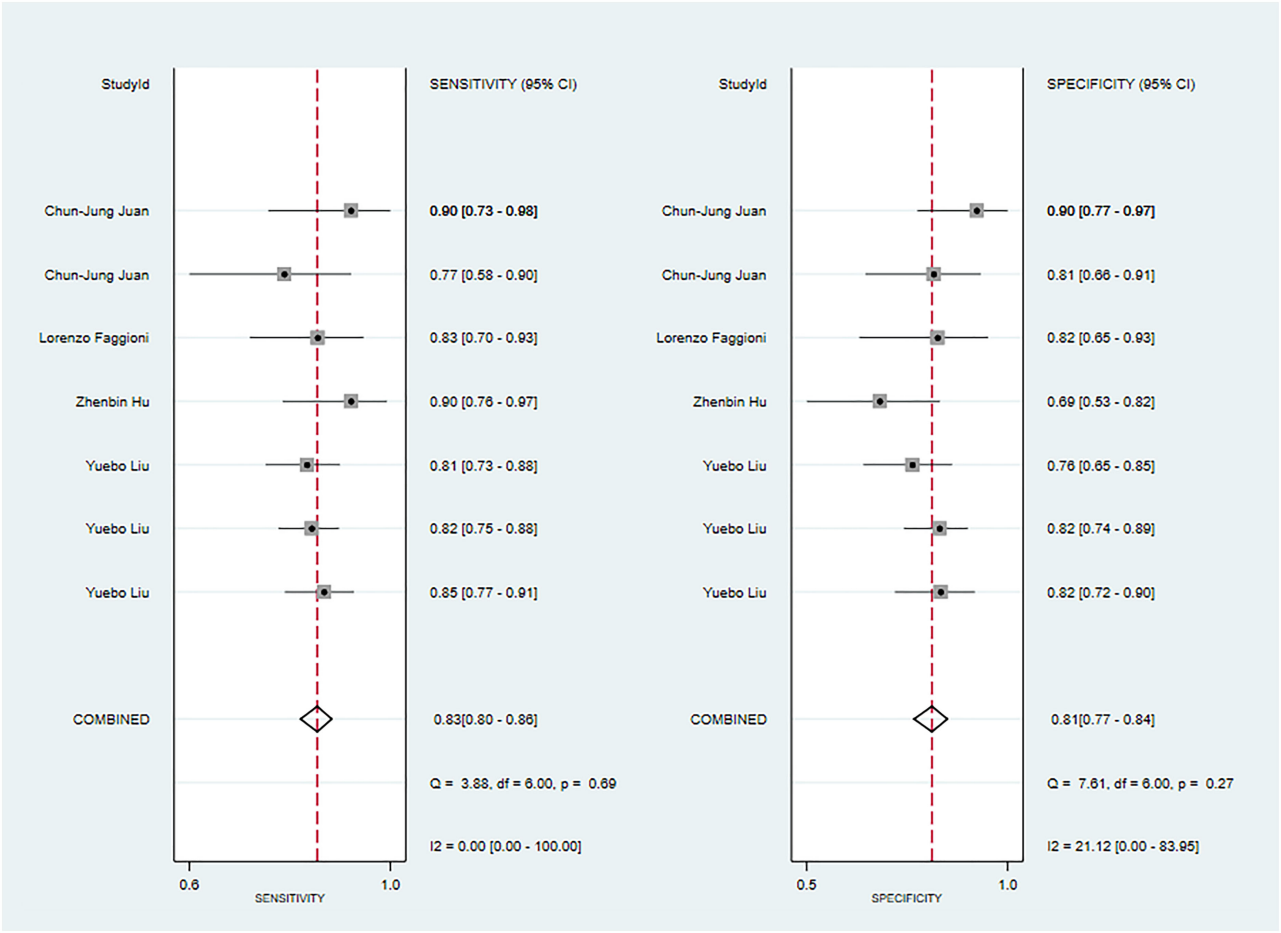
The Forest plot of radiomics based on MRI-based for the diagnosis of pleomorphic adenoma (The training set).

Four studies reported MRI-based models for the diagnosis of pleomorphic adenoma (50, 58, 62, 64). In the training set, MRI-based models had a sensitivity of 0.83[95%CI:0.80,0.86], a specificity of 0.81[95%CI:0.77~0.84], a PLR of 4.3[95%CI:3.5,5.2], an NLR of 0.21[95%CI:0.17,0.25], a DOR of 21[95%CI:15,29], and a SROC of 0.89[95%CI:0.86-0.91] (**Figure 11**; **Supplementary Figure S43**). Deek's funnel plot indicated no evidence of bias between models (P). With the prevalence of pleomorphic adenoma estimated in the included studies as the prior probability of the diagnostic experiment, the actual probability of pleomorphic adenoma was 84% if the model identified a pleomorphic adenoma (**Supplementary Figures S44**, **S45**).

##### 3.4.2.3 Clinical features

**Figure 12:**
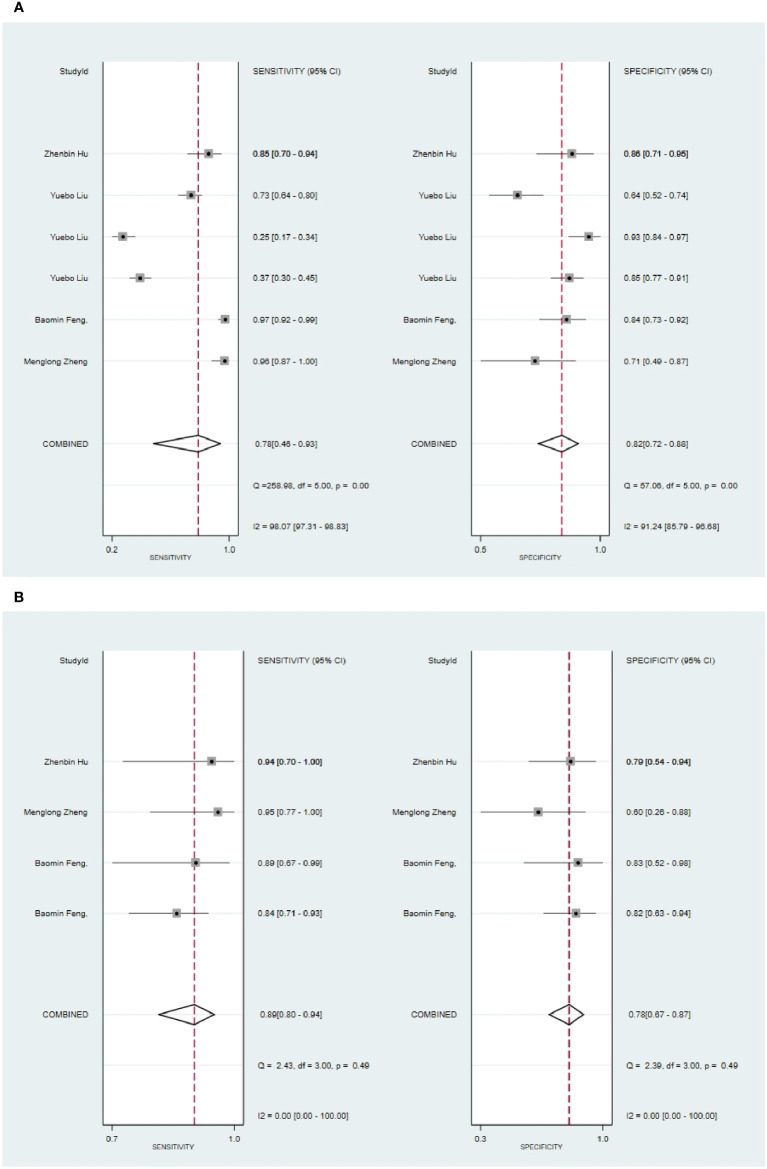
The Forest plot based on clinical features for the diagnosis of pleomorphic adenoma. **(A)** The training set; **(B)** The validation set.

Six primary studies reported models based on clinical features for the diagnosis of malignant tumors of the parotid gland (46, 51, 58, 64, 66, 68), and the machine learning model relying on clinical features alone demonstrated a sensitivity of 0.78[95%CI:0.46,0.93], a specificity of 0.82[95%CI:0.72,0.88], a PLR of 4.2[95%CI:2.8,6.4], an NLR of 0.27[95%CI:0.09,0.78], a DOR of16[95%CI:4,56], and a SROC of 0.86 [95%CI:0.82-0.88] (**Figure 12A**; **Supplementary Figure S46**). Deek's funnel plot indicated no evidence of publication bias between models (P). With the prevalence of pleomorphic adenoma estimated in the included studies as the prior probability of the diagnostic experiment, the actual probability of pleomorphic adenoma was 86% if the model identified a pleomorphic adenoma (**Supplementary Figures S47**, **S48**). In the validation set, five studies based on clinical features for the diagnosis of pleomorphic adenoma had a sensitivity of 0.89[95%CI: 0.80,0.94], a specificity of 0.78[95%CI:0.67,0.87], a PLR of 4.1[95%CI:2.6,6.5], an NLR of 0.14[95%CI:0.07,0.27], a DOR of 29[95%CI:12,71], and a SROC of 0.90 [95%CI:0.87-0.92] (**Figure 12B**; **Supplementary Figure S49**). Deek’s funnel plot indicated no evidence of publication bias between models (P). With the prevalence of pleomorphic adenoma estimated in the included studies as the prior probability of the diagnostic experiment, the actual probability of pleomorphic adenoma was 86% if the model identified a pleomorphic adenoma (**Supplementary Figures S50**, **S51**) (46, 49, 64).

##### 3.4.2.4 Integration of radiomic features with clinical features

**Figure 13:**
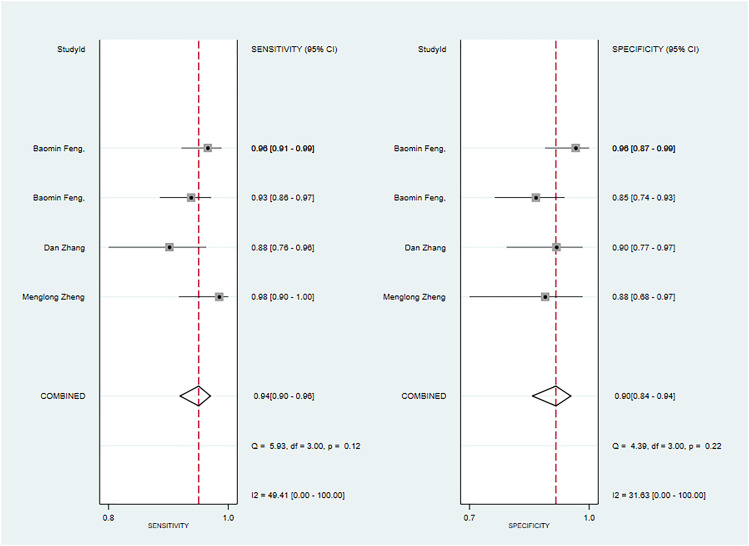
The Forest plot based on CT-based models combined with clinical features for the diagnosis of pleomorphic adenoma. (The training set).

Three primary studies reported CT-based models combined with clinical features for the diagnosis of pleomorphic adenoma (46, 49, 51). The machine learning model, incorporating radiomic features alongside clinical attributes, demonstrated a sensitivity of 0.94 [95%CI: 0.90, 0.96], a specificity of 0.90[95%CI:0.84, 0.94], a PLR of 9.7 [95%CI: 5.8,16.3], an NLR of 0.07[95%CI:0.04,0.11], a DOR of 148[95%CI:62, 352], and an SROC of 0.97[95%CI:0.95-0.98] (**Figure 13**; **Supplementary Figure S52**). Deek's funnel plot revealed no evidence of publication bias between models (P). With the prevalence of malignant parotid tumor estimated in the included studies as the prior probability of the diagnostic experiment, the actual probability of malignant parotid tumor was 94% if the model identified a malignant parotid tumor (**Supplementary Figures S53**, **S54**).

## 4 Discussion

### 4.1 Summary of the main findings

The validation set results reveal that the CT-based model exhibited a sensitivity of 0.80 [95% CI: 0.74 - 0.86] and a specificity of 0.84 [95% CI: 0.78 - 0.88] for detecting parotid tumors, predominantly malignant parotid tumors. In contrast, the MRI-based models demonstrated a sensitivity of 0.83[95% CI: 0.76 - 0.88] and a specificity of 0.82[95% CI: 0.77 - 0.86], indicating a commendable diagnostic performance. It is worth mentioning that the focus of existing studies on benign tumors, particularly Warthin’s tumor and pleomorphic adenoma, has been limited. However, it is essential to approach the findings concerning these tumors with caution due to the small number of primary studies examining them.

### 4.2 Comparison with previous reviews

This systematic review highlights the potential of radiomics in distinguishing between benign and malignant parotid tumors. Moreover, both invasive and non-invasive methods are utilized to differentiate between the two. C. Carrie Liu et al. (70) presented supporting evidence for the use of ultrasound-guided Fine Needle Aspiration (FNA) as a means of differentiation. Their findings indicate that FNA has a sensitivity of 0.882 (95% CI: 0.509-0.982) and a specificity of 0.995 (95% CI: 0.960-0.999) in this regard. Hee Joon Kim (71) provided evidence for the use of ultrasound-guided Core Needle Biopsy (CNB) as a method for discriminating between benign and malignant parotid tumors. The reported sensitivity and specificity of CNB were 0.94 (95% CI: 0.92-0.96) and 0.98 (95% CI: 0.97-0.99), respectively. Yun-Fei Zhang et al. (72) supported the use of ultrasound elastography in distinguishing between the two types of tumors. The reported sensitivity and specificity were 0.67 (95% CI: 0.59-0.74) and 0.64 (95% CI: 0.60-0.68), respectively. Jing Chen et al. (73) provided further evidence by calculating the ADC value through diffusion-weighted imaging (DWI) for discriminating between benign and malignant parotid tumors, with a reported sensitivity of 0.89 (95% CI: 0.82-0.93) and specificity of 0.76 (95% CI: 0.67-0.83).

In their study, Ying-Ying Liang et al. (74) presented evidence-based findings on differentiating between benign and malignant parotid tumors using various MRI techniques. The results demonstrated that conventional MRI, diffusion-weighted imaging (DWI), dynamic contrast-enhanced scanning (DCE), and their combined examination had sensitivities and specificities of 0.76 [95% CI: 0.63-0.86]/0.91 [95% CI: 0.81-0.97]/0.80 [95% CI: 0.70-0] and 0.83 [95% CI: 0.77-0.88]/0.56 [95% CI: 0.47-0.64]/0.90[95% CI: 0.86-0.94]/0.90 [95% CI: 0.85-0.94], respectively. Our data revealed that CT-based models had a sensitivity of 0.80[95% CI: 0.73-0.85]and a specificity of 0.85 [95% CI: 0.80-0.89] in discriminating between benign and malignant parotid tumors, particularly malignant tumors of the parotid gland. In the validation set, CT-based models demonstrated a sensitivity of 0.83 [95% CI: 0.76-0.88] and a specificity of 0.81 [95% CI: 0.77-0.85]. Furthermore, the accuracy of CT or MRI-based models surpassed that of ultrasound elastography. Compared to the ADC values calculated by DWI, CT or MRI-based models exhibited a higher specificity and a lower sensitivity. Although the accuracy of CT- and MRI-based models exceeded that of conventional MRI, it was lower than that of DCE. DWI-based models possessed a higher sensitivity but a lower specificity compared to CT- and MRI-based radiomics. The accuracy of FNA and CNB was higher than that of CT- and MRI-based models. Additionally, Roie Fisher (69) provided evidence-based support for FNA in identifying Warthin’s tumor, with a sensitivity and specificity of 93.7% [95% CI: 92.1-95.3] and 97.9% [95% CI: 97.9-98.9], respectively. This accuracy surpassed that of MRI-based models in our study. These findings suggest that existing radiomics is not significantly superior to conventional invasive or noninvasive diagnostic methods in disease diagnosis. Therefore, further studies are necessary to improve the diagnostic accuracy. At the same time, the diagnostic advantage of radiomics should not be overlooked. The aforementioned studies primarily rely on laboratory methods or radiologist diagnosis, which are inefficient and have limited ability in early differential diagnosis between benign and malignant parotid tumors. Radiomics, however, offers a fast and less error-prone approach to disease diagnosis compared to conventional methods, contributing to the progress of radiomics.

Our included studies have revealed that CT-based radiomics demonstrates excellent performance in distinguishing between malignant parotid tumors and pleomorphic adenoma (PA). Its accuracy is comparable to that of MRI, with no significant inferiority observed. It is worth noting that PA, which accounts for 65% of all parotid tumors, is considered a borderline tumor. Moreover, studies pertaining to this topic suggest that there is a 5% or higher risk of malignant transformation associated with PA (75), and incomplete removal of PA also carries the risk of local recurrence. It has been reported that the recurrence rate of patients undergoing PA extraction is higher than that of patients undergoing parotidectomy (76), so preoperative differential diagnosis of PA is necessary. However, in the field of clinical practice, the utilization of MRI is constrained by its relatively high cost and lower level of patient acceptance in comparison to CT. Hence, it would be prudent to consider the development of a more cost-effective and widely accepted differentiation method based on CT in future research endeavors.

The RQS scores for the included studies are concerning, suggesting potential high bias in these investigations. Therefore, it is important to conduct a comprehensive evaluation of the RQS scale, as certain criteria within the scale seem excessively strict and may not be in line with current practices in radiomics research. Given these circumstances, a careful scrutiny of the RQS scale is warranted.

Before initiating the research, it is necessary to adjust and compare the parameters under different equipment. Alternatively, conducting multiple imaging sessions at different times after starting the study would also be beneficial. The majority of the included studies utilized retrospective designs, which poses challenges in ensuring adherence to the scale requirements. Furthermore, most of these studies were not publicly registered, even though registered scores constitute a substantial portion of the overall scale. Many primary studies received zero scores due to their primarily single-center nature, which makes it difficult to achieve independent external validation. Moreover, plotting decision curves and clinical impact curves for CNN and SVM models is challenging under the current conditions, further hindering their scores.

### 4.3 Strengths and limitations of the study

This study provides evidence-based insights into the diagnosis and distinction of benign and malignant parotid tumors using radiomics, marking a significant contribution to the field. It serves as a valuable reference for the advancement of radiomics in this particular area. However, it is important to note that our study does have certain limitations. Firstly, while there is a diverse range of radiomic methods available, they have not been extensively explored in our research. Secondly, the number of studies on Warthin’s tumor and pleomorphic adenoma is relatively small, which restricts the scope of our findings.

## 5 Conclusions

Models based on radiomics have the potential to improve the accuracy of distinguishing between benign and malignant parotid tumors. This can provide clinicians with personalized, non-invasive predictive methods before surgery, allowing for valuable predictions and facilitating better treatment strategies for patients. In future studies, it is important to utilize standardized radiomics-based features, more reliable feature selection methods, and advanced model development tools to further enhance the diagnostic accuracy of AI in differentiating between benign and malignant parotid gland tumors.

## Data availability statement

The original contributions presented in the study are included in the article/**Supplementary Material**. Further inquiries can be directed to the corresponding authors.

## Author contributions

YR: Conceptualization, Formal analysis, Investigation, Methodology, Writing – original draft, Writing – review & editing. JHW: Conceptualization, Formal analysis, Investigation, Methodology, Writing – review & editing. WX: Conceptualization, Formal analysis, Investigation, Methodology, Writing – review & editing. YM: Conceptualization, Formal analysis, Investigation, Methodology, Writing – review & editing. JQW: Conceptualization, Formal analysis, Investigation, Methodology, Writing – review & editing. LS: Conceptualization, Formal analysis, Investigation, Methodology, Writing – review & editing. LG: Conceptualization, Formal analysis, Funding acquisition, Investigation, Methodology, Resources, Supervision, Writing – review & editing. LF: Conceptualization, Formal analysis, Funding acquisition, Investigation, Methodology, Resources, Supervision, Writing – review & editing.
