# Supplementary material for: Performance of radiomics in the differential diagnosis of parotid tumors: a systematic review
Source: Front Oncol. 2024 Jul 25;14:1383323. doi: 10.3389/fonc.2024.1383323 (PMC11306159; doi:10.3389/fonc.2024.1383323)
Supplement: Supplementary file 1 [file DataSheet_1.zip › Supplementary figures-R1.docx]

**
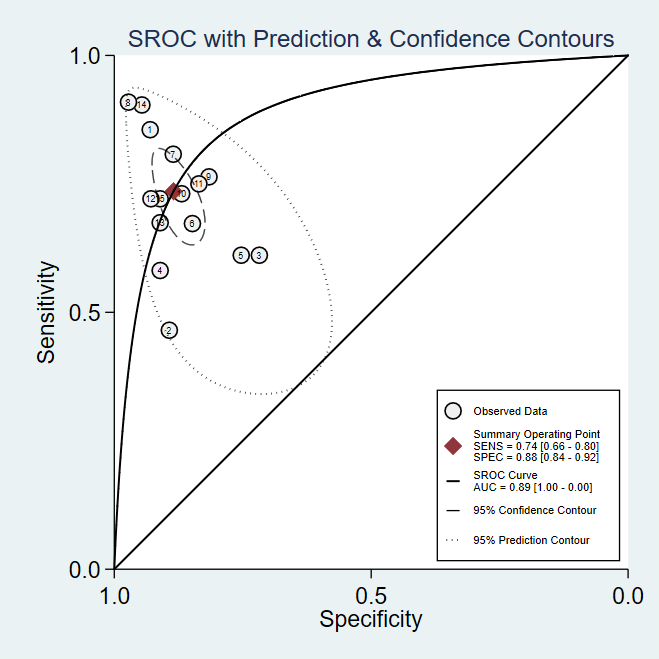
**

**Figure S1:** The SROC curve of radiomics based on CT for the diagnosis of malignant tumors (The training set)


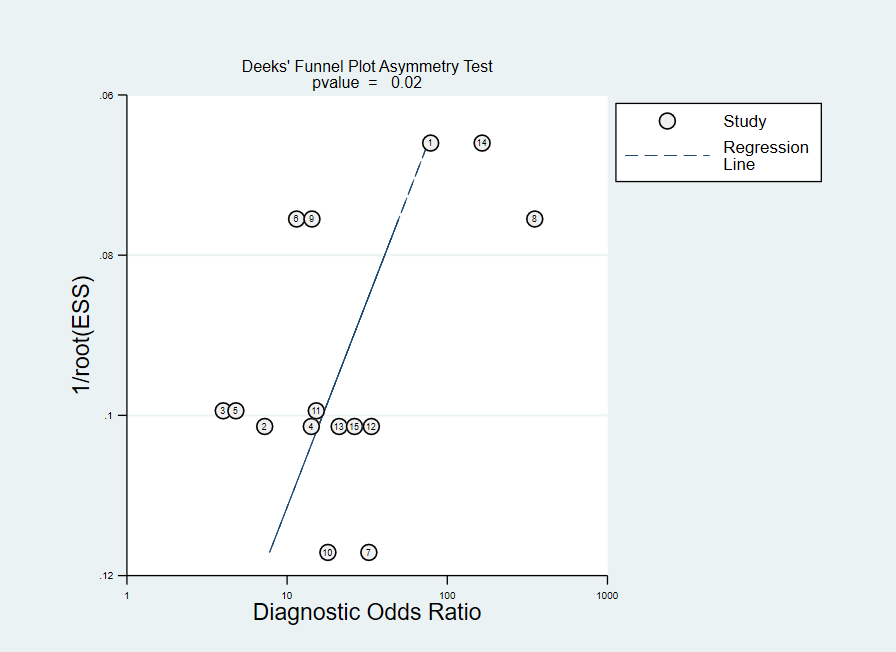


**Figure S2:** The Deek's funnel plot of radiomics based on CT for the diagnosis of malignant tumors (The training set)


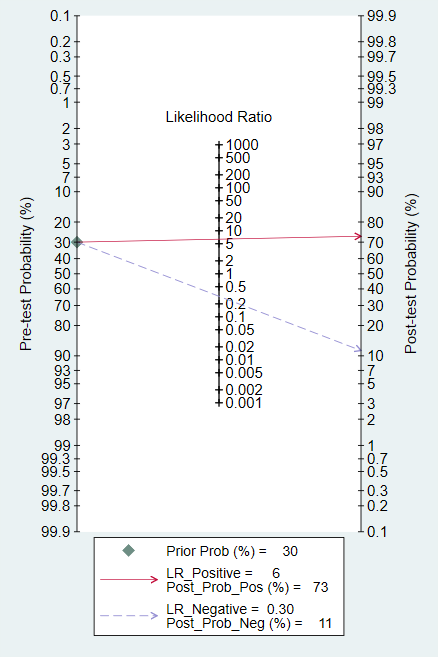


**Figure S3:** The Deek's funnel plot of radiomics based on CT for the diagnosis of malignant tumors (The training set)


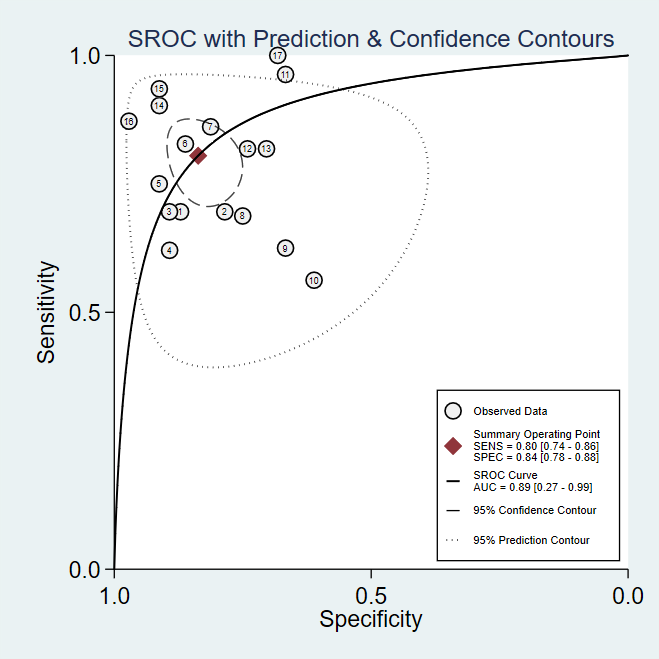


**Figure S4:** The SROC curve of radiomics based on CT for the diagnosis of malignant tumors (The validation set)


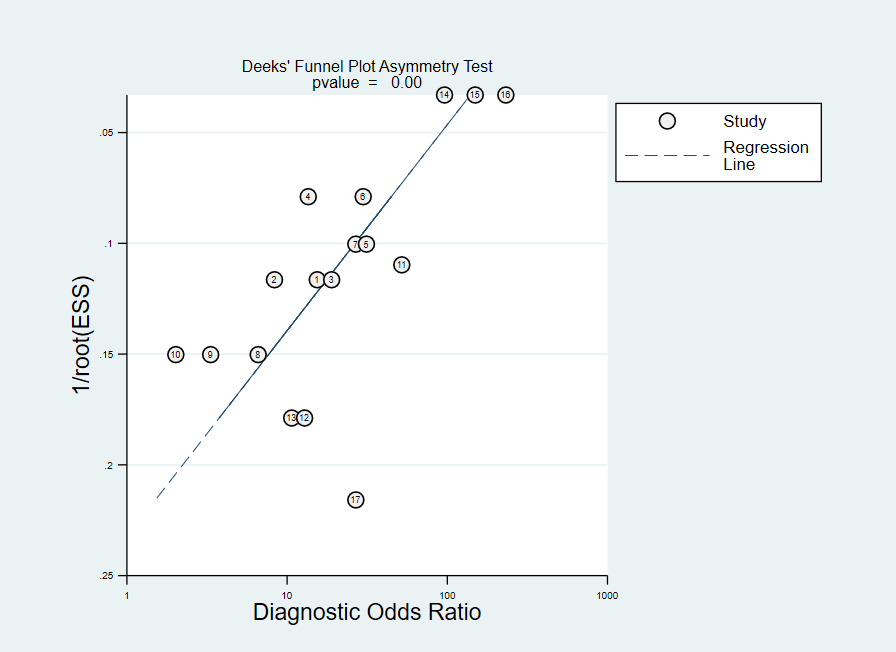


**Figure S5:** The Deek's funnel plot of radiomics based on CT for the diagnosis of malignant tumors (The validation set)


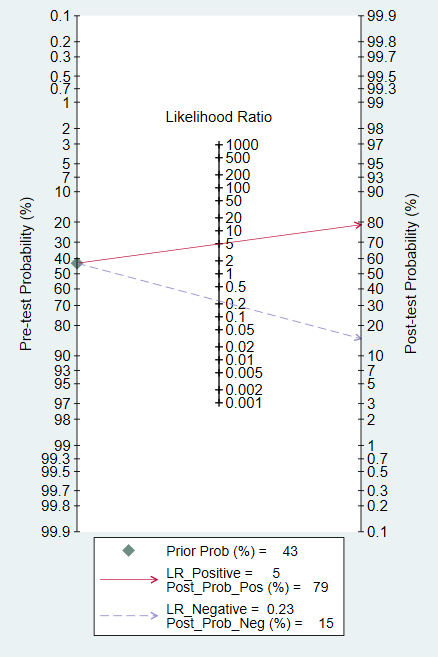


**Figure S6:** The Deek's funnel plot of radiomics based on CT for the diagnosis of malignant tumors (The validation set)


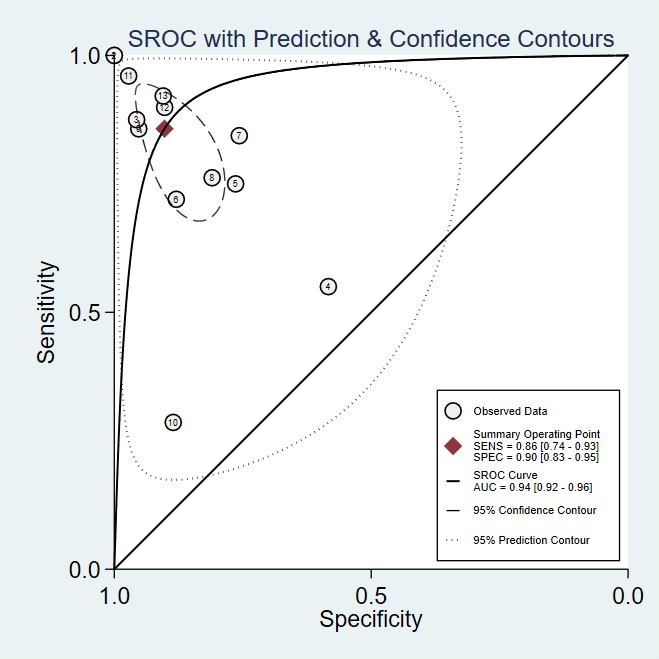


**Figure S7:** The SROC curve of radiomics based on MRI for the diagnosis of malignant tumors (The training set)


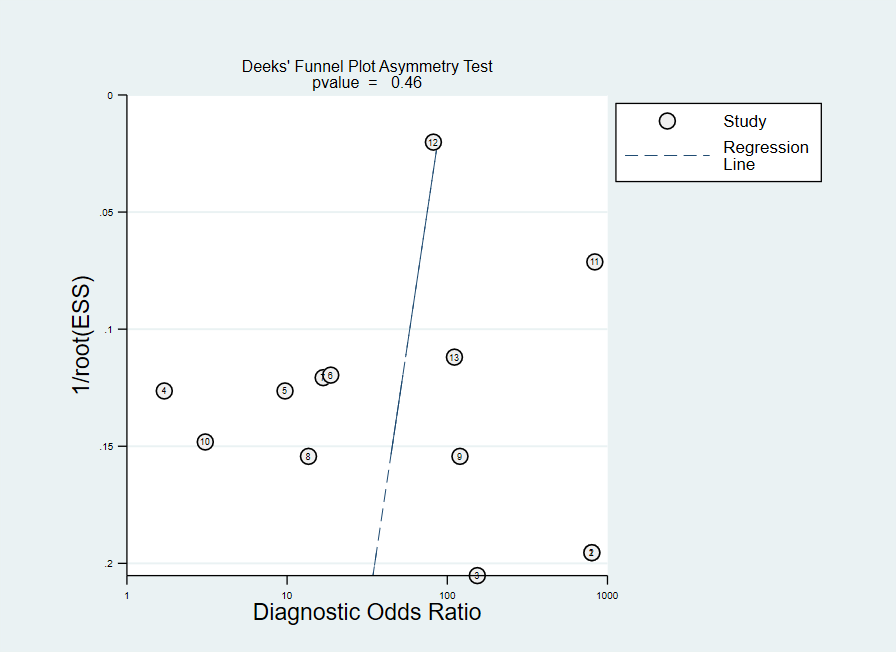


**Figure S8:** The Deek's funnel plot of radiomics based on MRI for the diagnosis of malignant tumors (The training set)


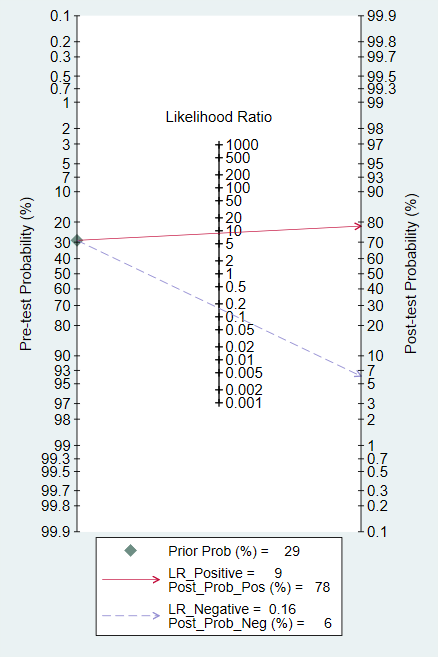


**Figure S9:** The Deek's funnel plot of radiomics based on MRI for the diagnosis of malignant tumors (The training set)


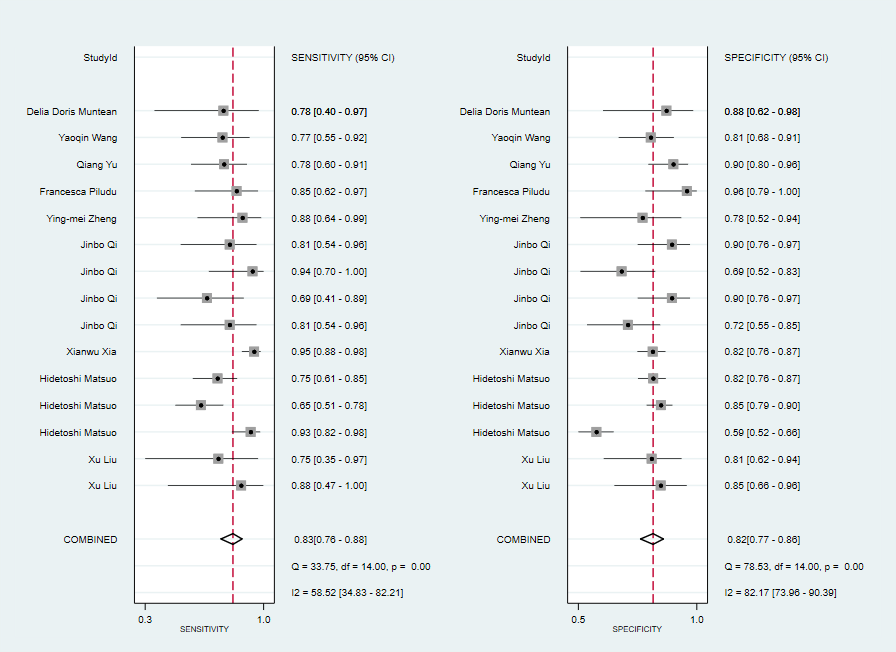


**Figure S10:** The SROC curve of radiomics based on MRI for the diagnosis of malignant tumors (The validation set)


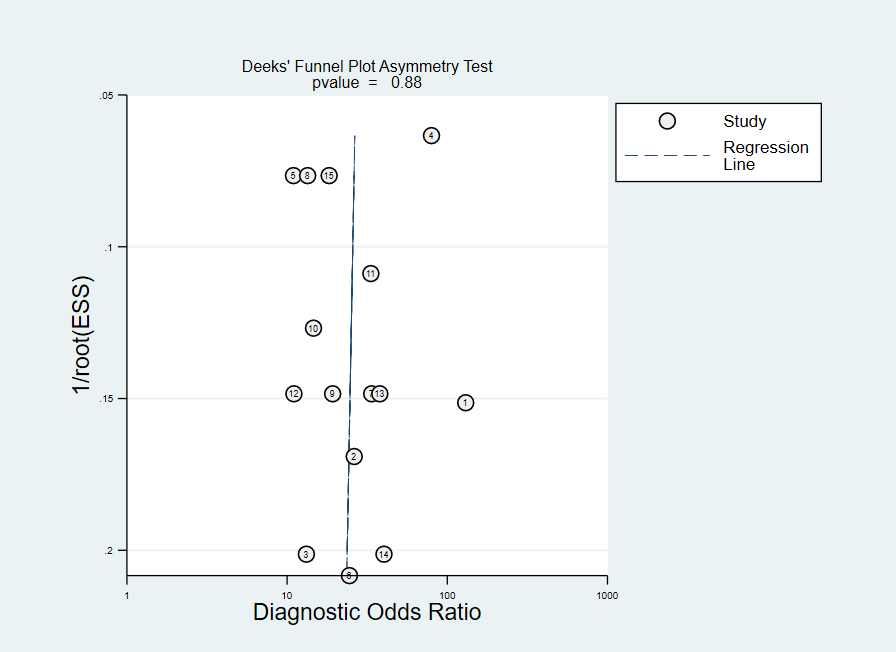


**Figure S11:** The Deek's funnel plot of radiomics based on MRI for the diagnosis of malignant tumors (The validation set)


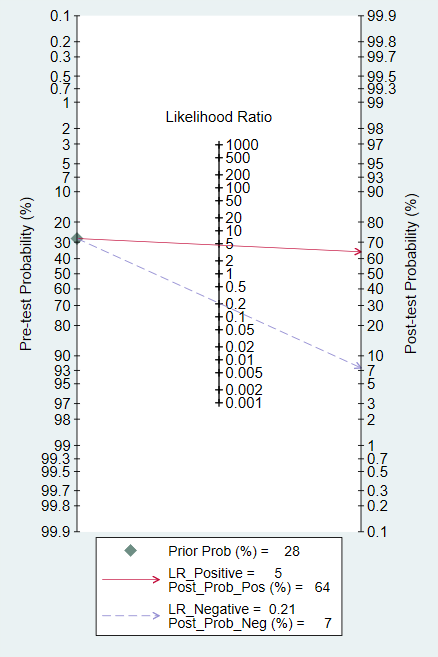


**Figure S12:** The Deek's funnel plot of radiomics based on MRI for the diagnosis of malignant tumors (The validation set)


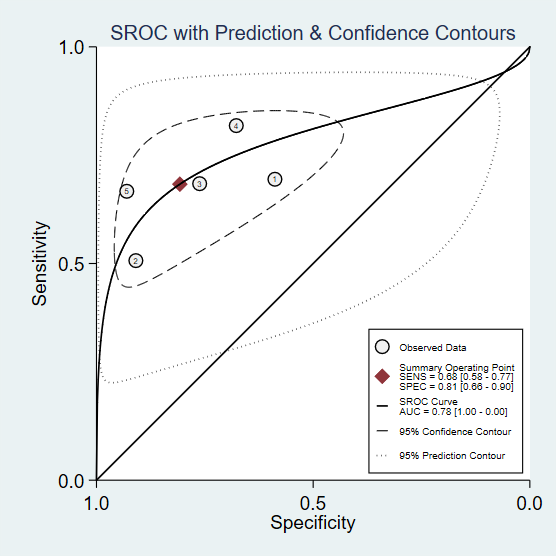


**Figure S13:** The SROC curve based on clinical features for the diagnosis of malignant tumors (The training set)


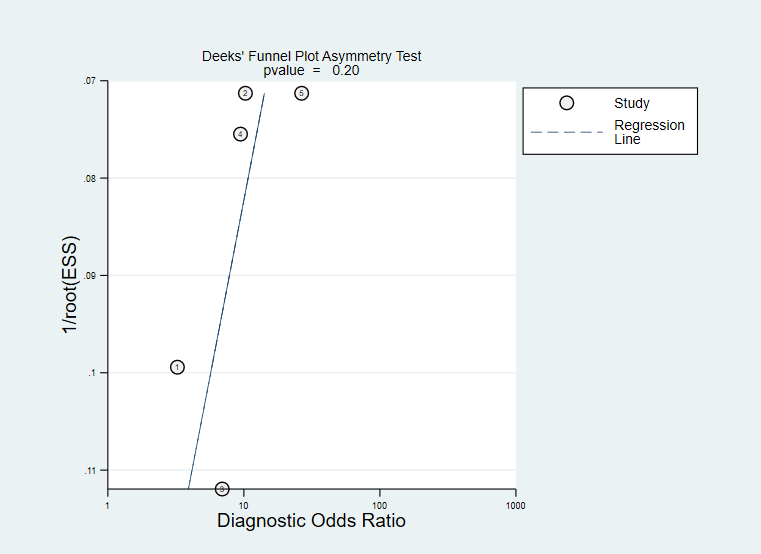


**Figure S14:** The Deek's funnel plot based on clinical features for the diagnosis of malignant tumors (The training set)


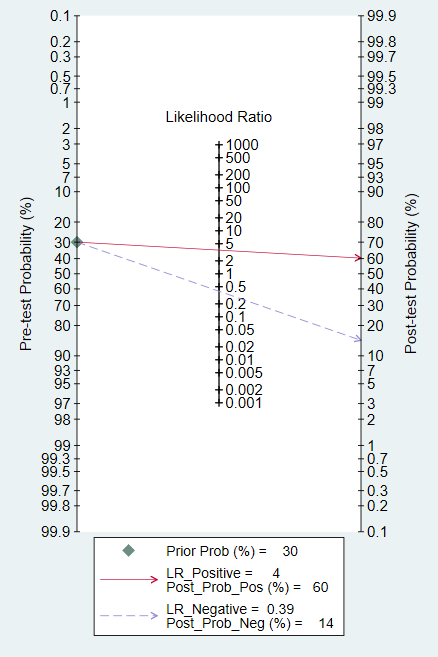


**Figure S15:** The Deek's funnel plot based on clinical features for the diagnosis of malignant tumors (The training set)


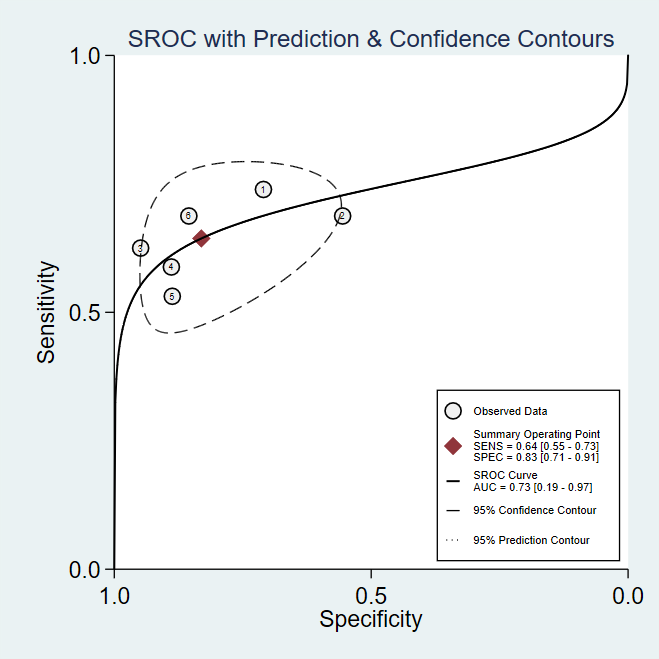


**Figure S16:** The SROC curve based on clinical features for the diagnosis of malignant tumors (The validation set)


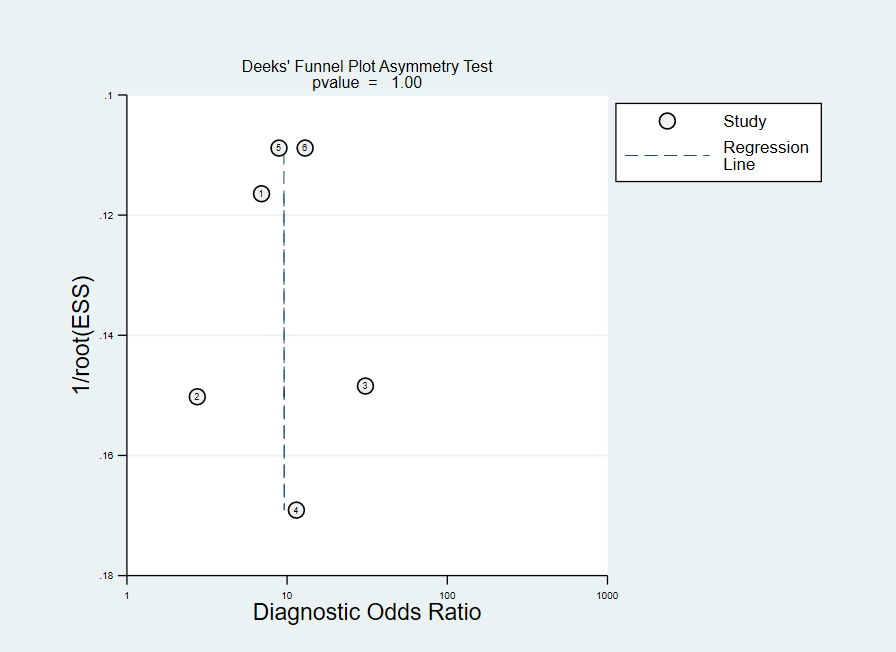


**Figure S17:** The Deek's funnel plot based on clinical features for the diagnosis of malignant tumors (The validation set)


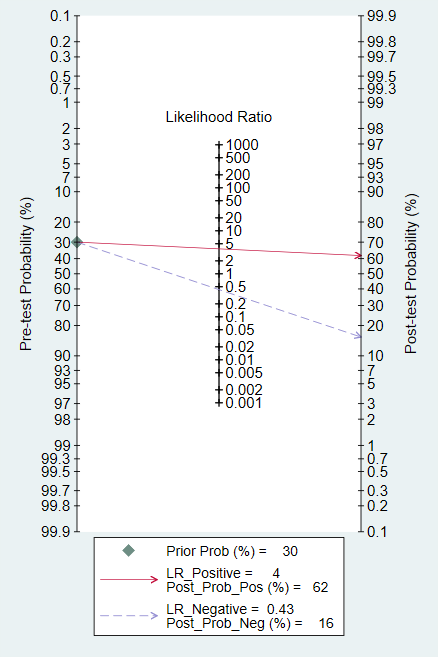


**Figure S18:** The Deek's funnel plot based on clinical features for the diagnosis of malignant tumors (The validation set)


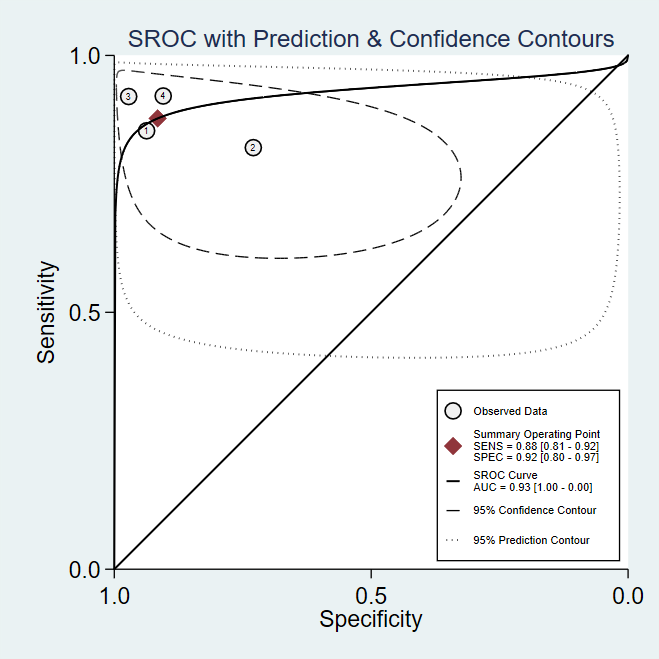


**Figure S19:** The SROC curve based on MRI-based models combined with clinical features for the diagnosis of malignant tumors (The validation set)


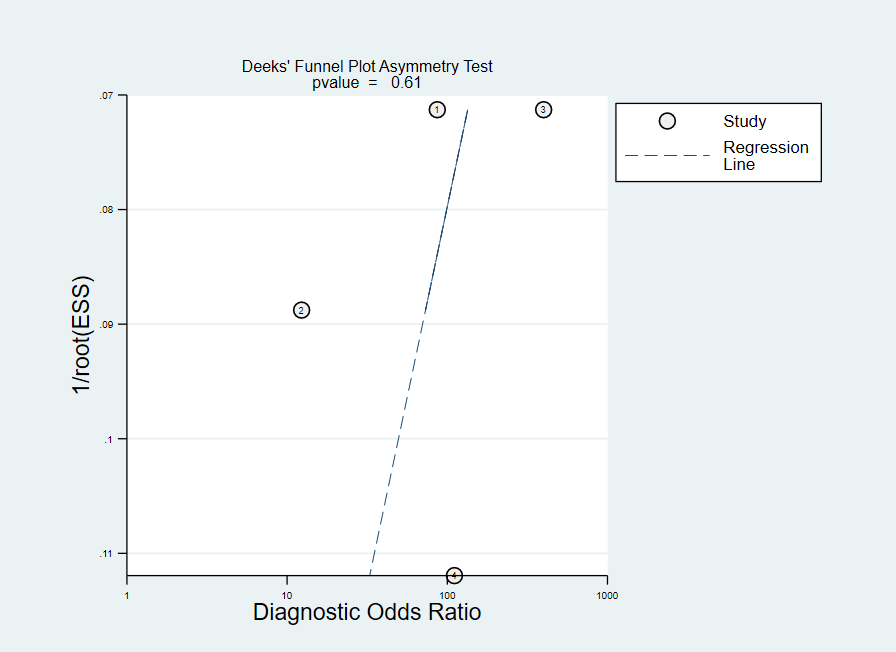


**Figure S20:** The Deek's funnel plot based on MRI-based models combined with clinical features for the diagnosis of malignant tumors (The training set)


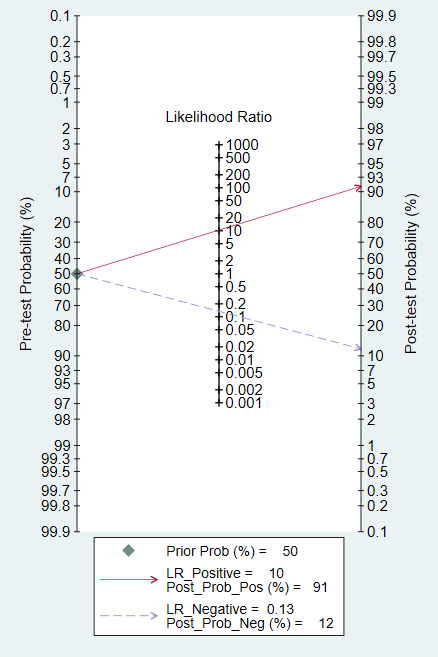


**Figure S21:** The Deek's funnel plot based on MRI-based models combined with clinical features for the diagnosis of malignant tumors (The training set)


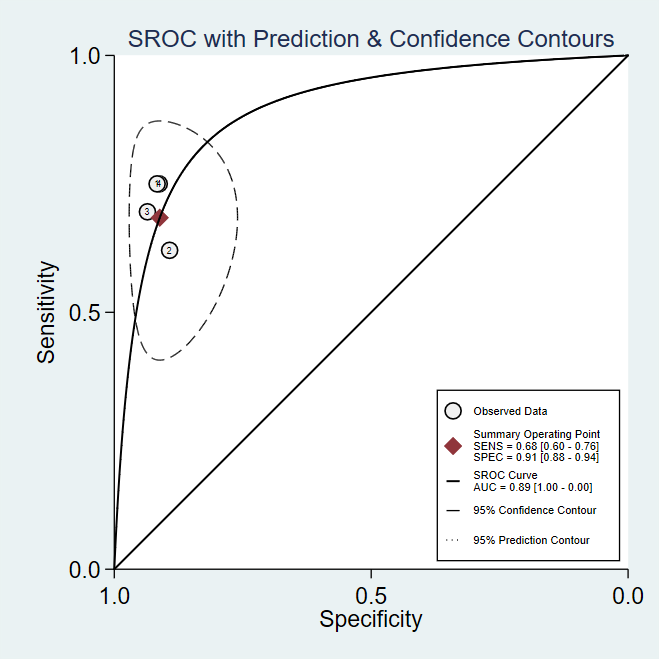


**Figure S22:** The SROC curve based on CT-based models combined with clinical features for the diagnosis of malignant tumors (The validation set)


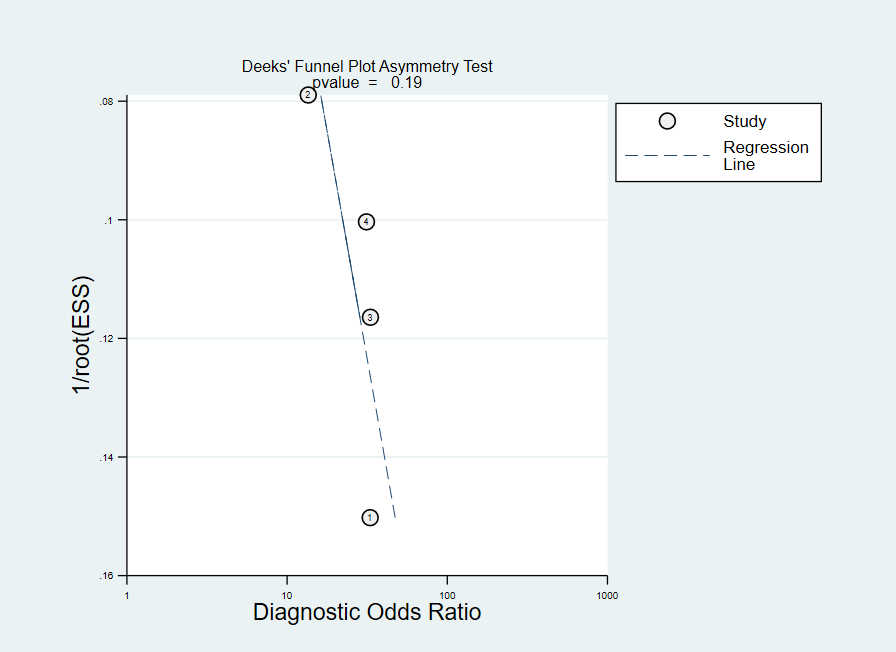


**Figure S23:** The Deek's funnel plot based on CT-based models combined with clinical features for the diagnosis of malignant tumors (The validation set)


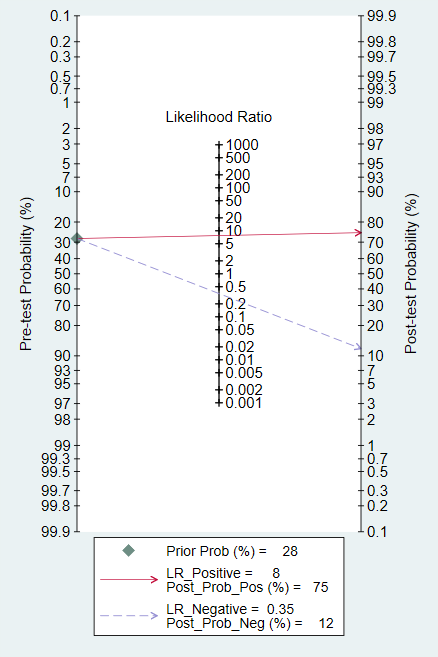


**Figure S24:** The Deek's funnel plot based on CT-based models combined with clinical features for the diagnosis of malignant tumors (The validation set)


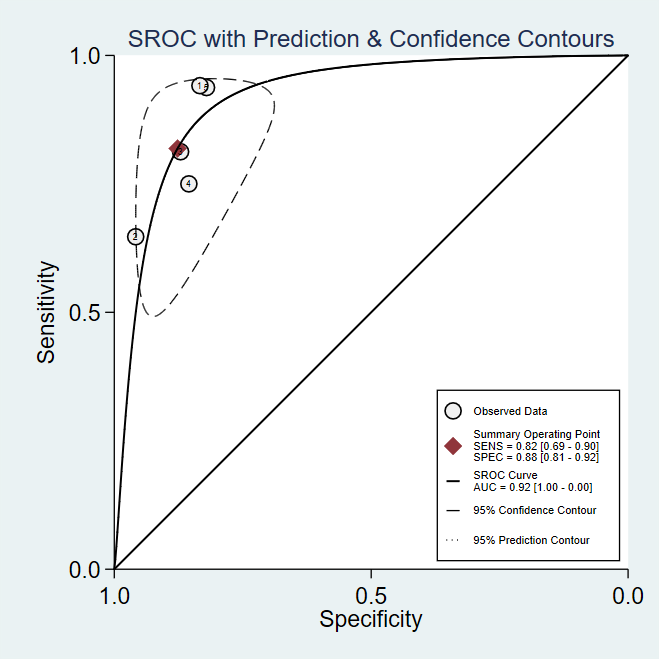


**Figure S25:** The SROC curve based on MRI-based models combined with clinical features for the diagnosis of malignant tumors (The validation set)


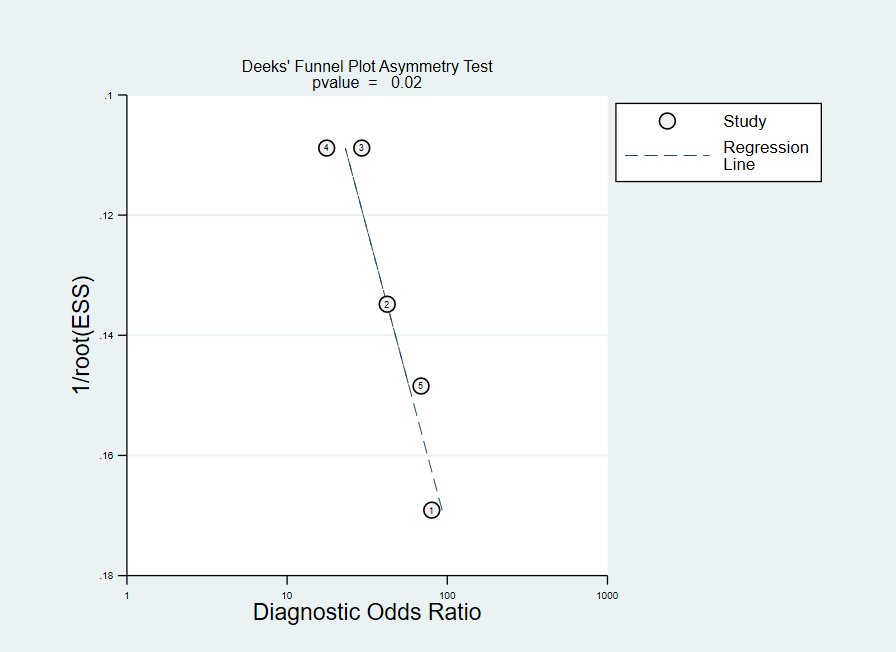


**Figure S26:** The Deek's funnel plot based on MRI-based models combined with clinical features for the diagnosis of malignant tumors (The validation set)


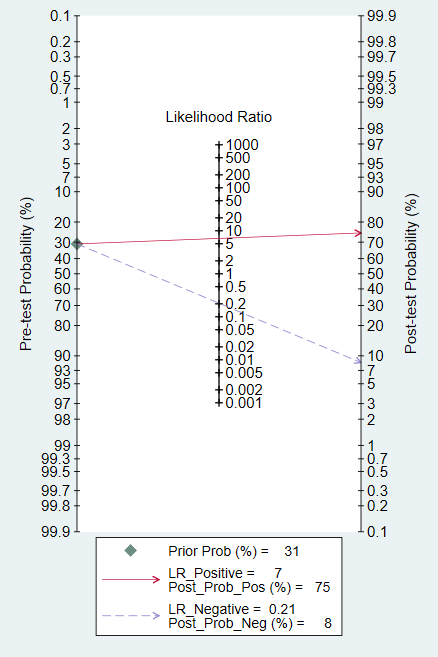


**Figure S27:** The Deek's funnel plot based on MRI-based models combined with clinical features for the diagnosis of malignant tumors (The validation set)


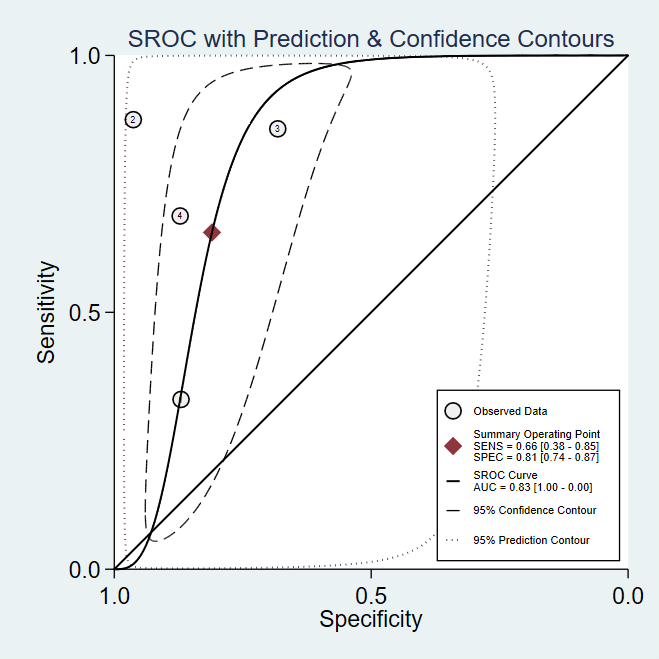


**Figure S28:** The SROC curve based on ADC values alone for the diagnosis of malignant tumors (The validation set)


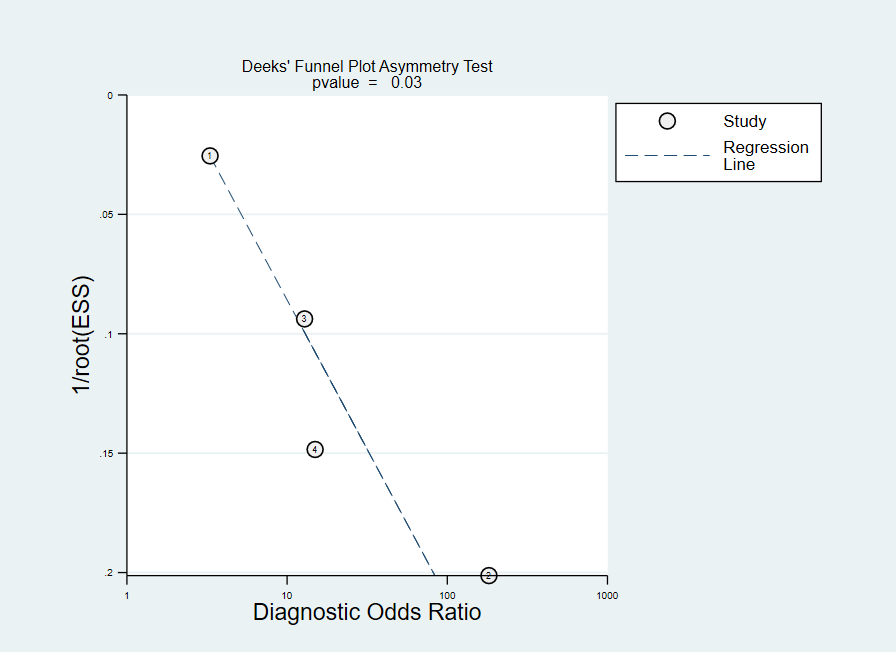


**Figure S29:** The Deek's funnel plot based on ADC values alone for the diagnosis of malignant tumors (The validation set)


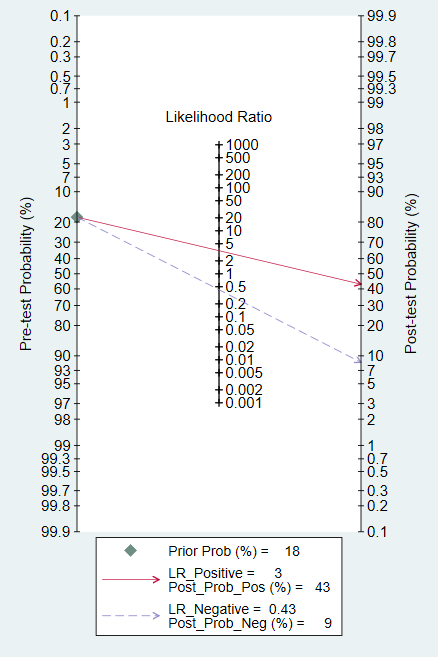


**Figure S30:** The Deek's funnel plot based on ADC values alone for the diagnosis of malignant tumors (The validation set)


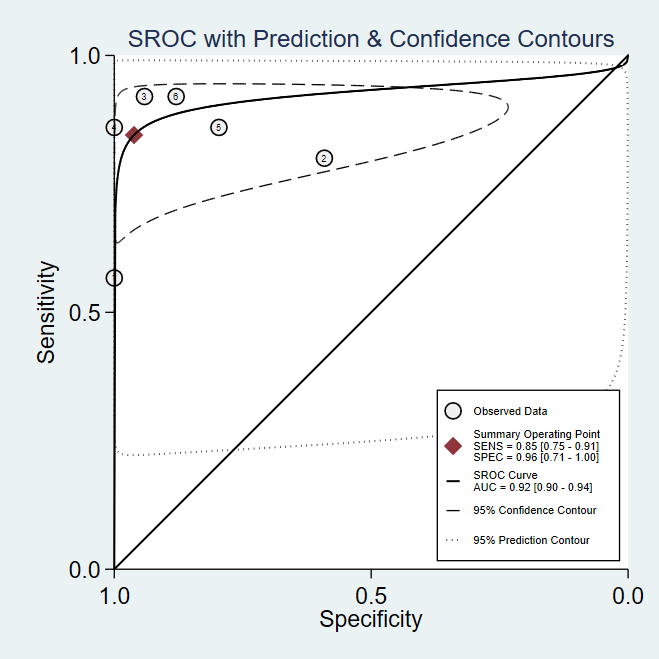


**Figure S31:** The SROC curve of radiomics based on CT-based models for the diagnosis of Warthin’s tumors (The validation set)


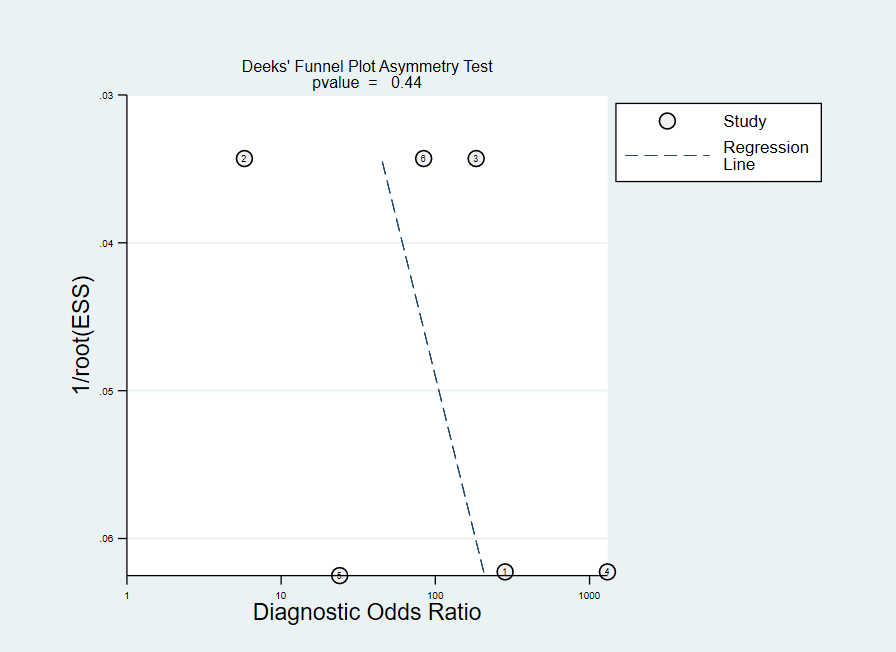


**Figure S32:** The Deek's funnel plot curve of radiomics based on CT-based models for the diagnosis of Warthin’s tumors (The validation set)


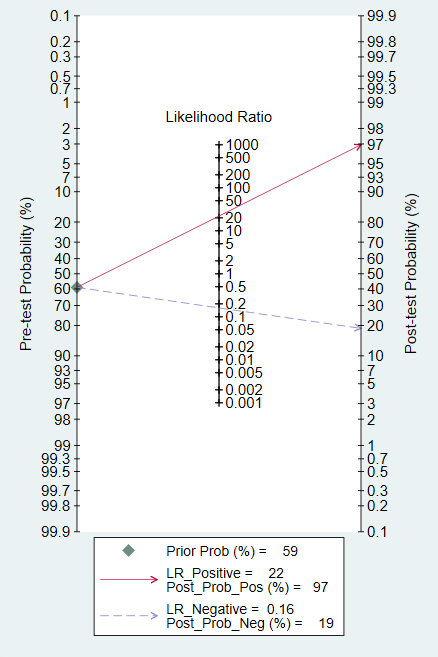


**Figure S33:** The Deek's funnel plot curve of radiomics based on CT-based models for the diagnosis of Warthin’s tumors (The validation set)


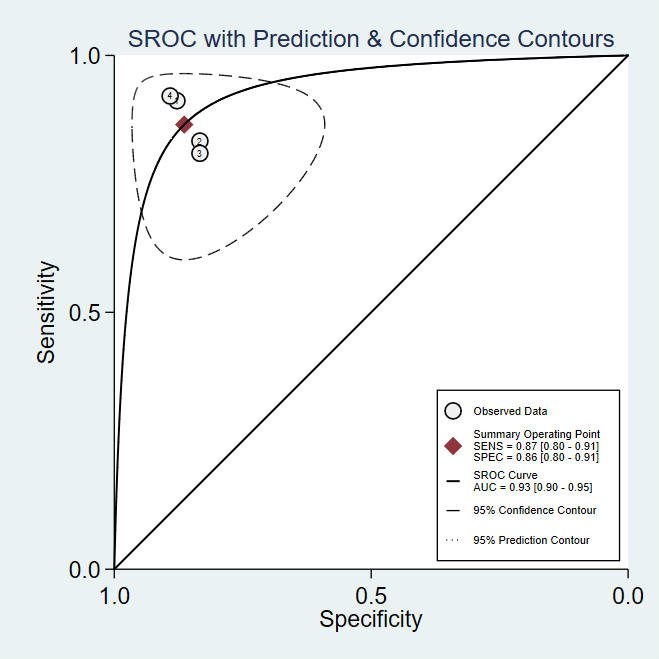


**Figure S34:** The SROC curve of radiomics based on MRI-based models for the diagnosis of Warthin’s tumors (The training set)


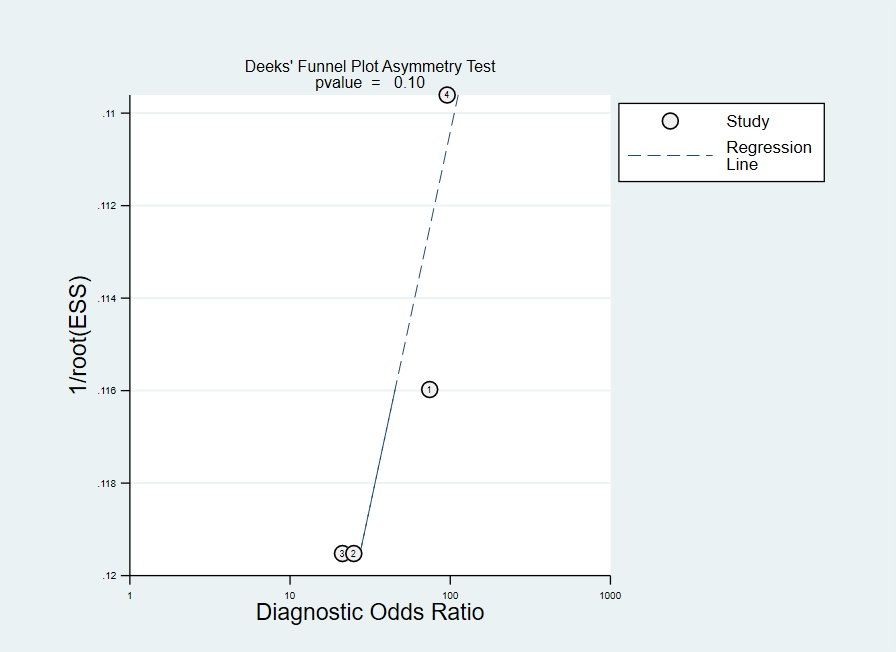


**Figure S35:** The Deek's funnel plot curve of radiomics based on MRI-based models for the diagnosis of Warthin’s tumors (The training set)


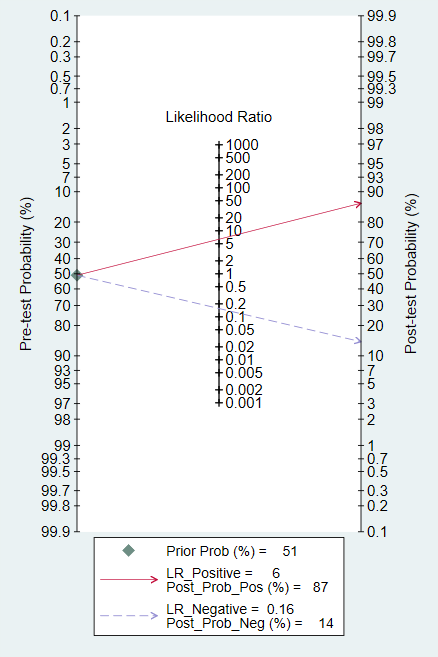


**Figure S36:** The Deek's funnel plot curve of radiomics based on MRI-based models for the diagnosis of Warthin’s tumors (The training set)


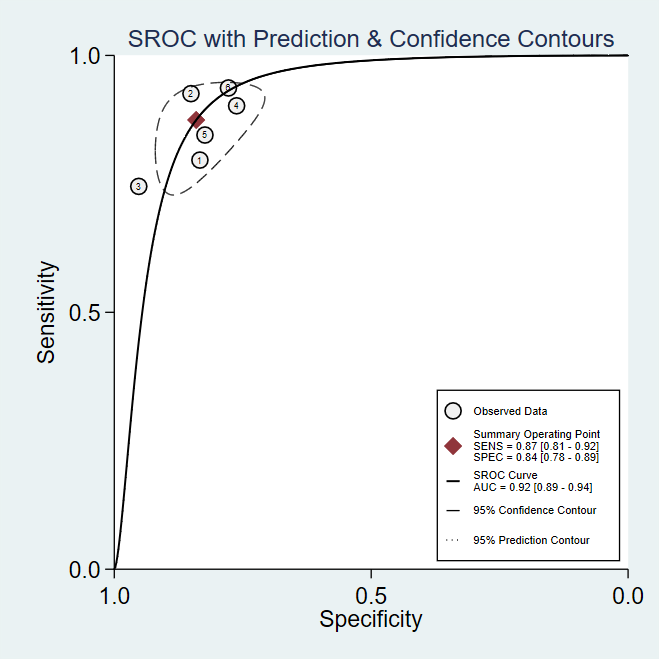


**Figure S37:** The SROC curve of radiomics based on CT-based models for the diagnosis of pleomorphic adenoma (The training set)


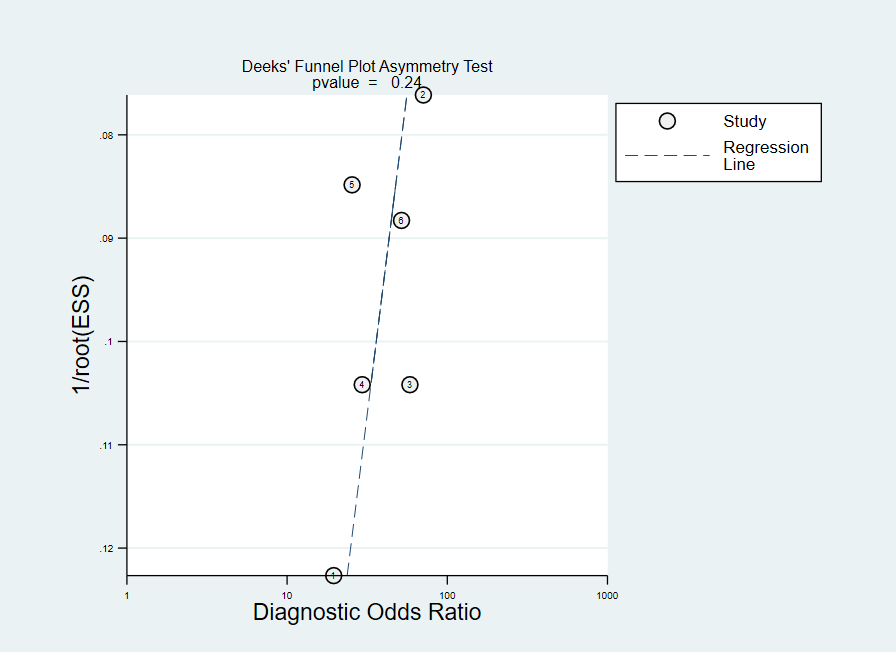


**Figure S38:** The Deek's funnel plot curve of radiomics based on CT-based models for the diagnosis of pleomorphic adenoma (The training set)


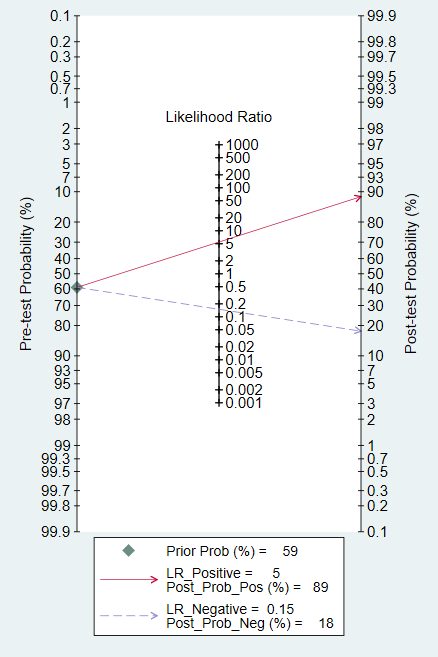


**Figure S39:** The Deek's funnel plot curve of radiomics based on CT-based models for the diagnosis of pleomorphic adenoma (The training set)


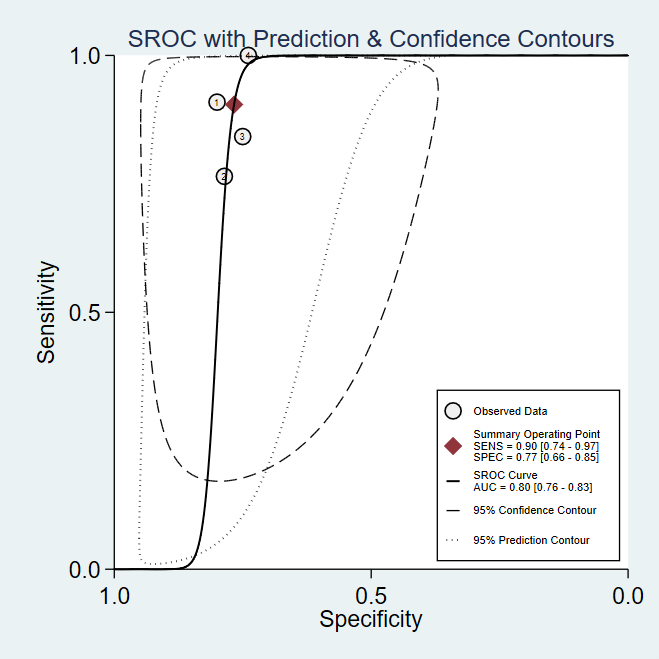


**Figure S40:** The SROC curve of radiomics based on CT-based models for the diagnosis of pleomorphic adenoma (The validation set)


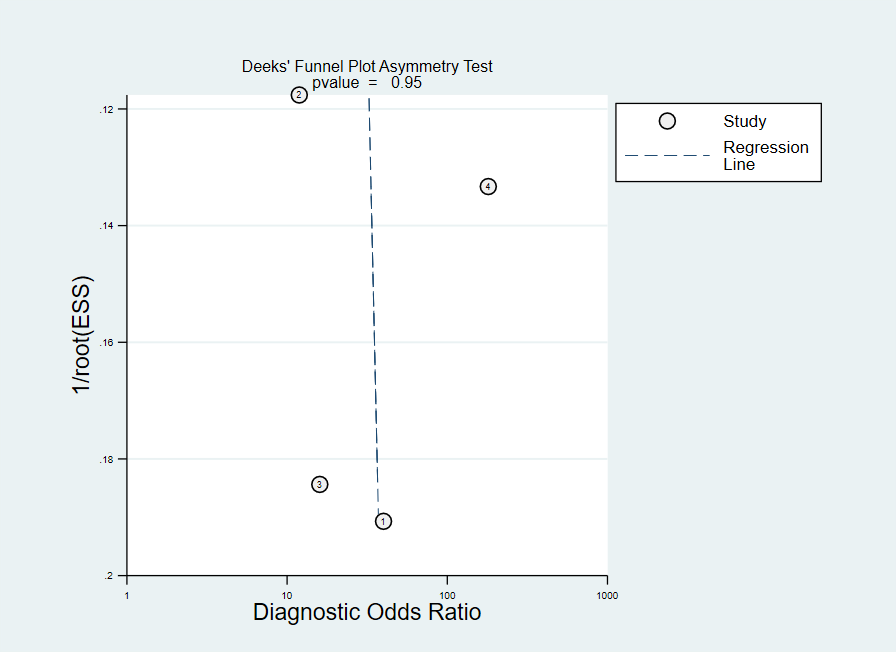


**Figure S41:** The Deek's funnel plot curve of radiomics based on CT-based models for the diagnosis of pleomorphic adenoma (The validation set)


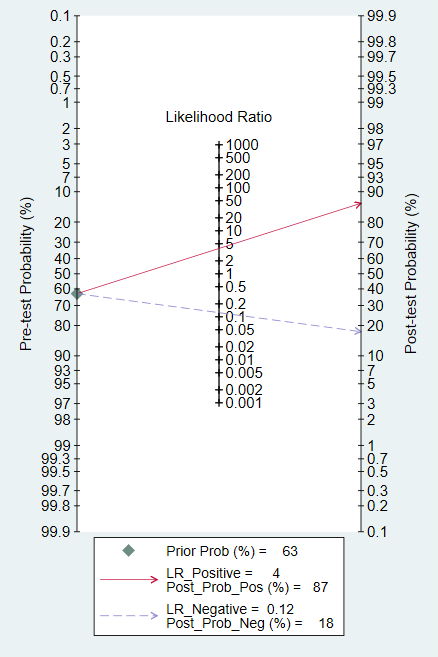


**Figure S42:** The Deek's funnel plot curve of radiomics based on CT-based models for the diagnosis of pleomorphic adenoma (The validation set)


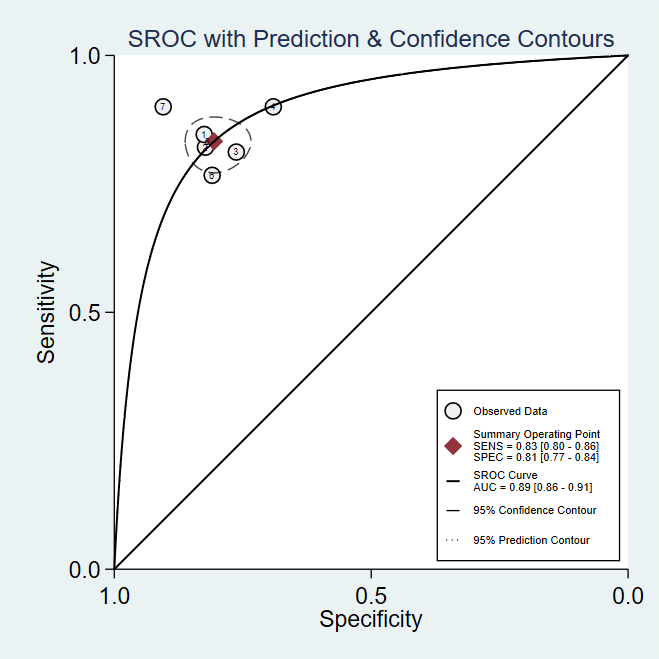


**Figure S43:** The SROC curve of radiomics based on MRI-based models for the diagnosis of pleomorphic adenoma (The training set)


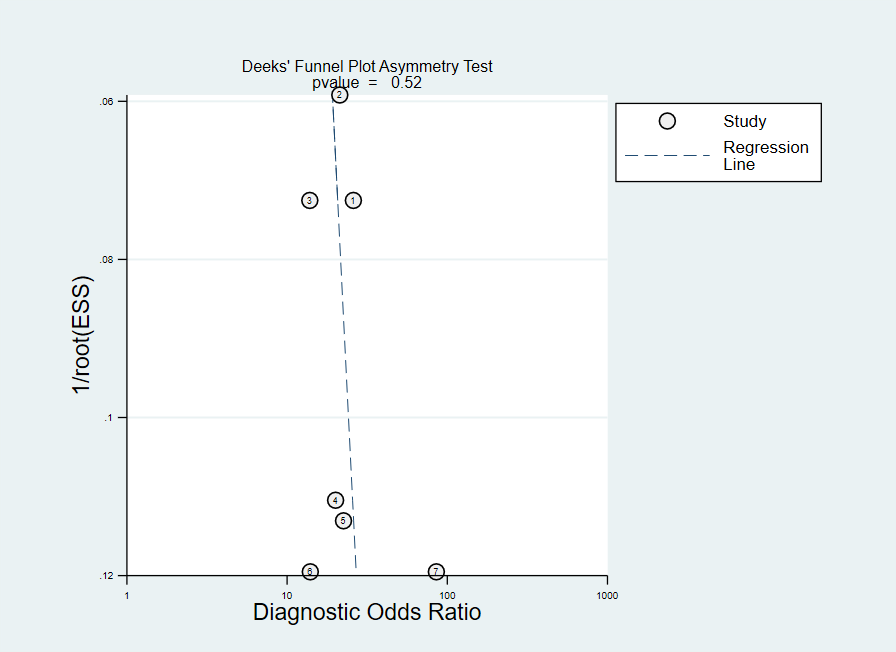


**Figure S44:** The Deek's funnel plot curve of radiomics based on MRI-based models for the diagnosis of pleomorphic adenoma (The training set)


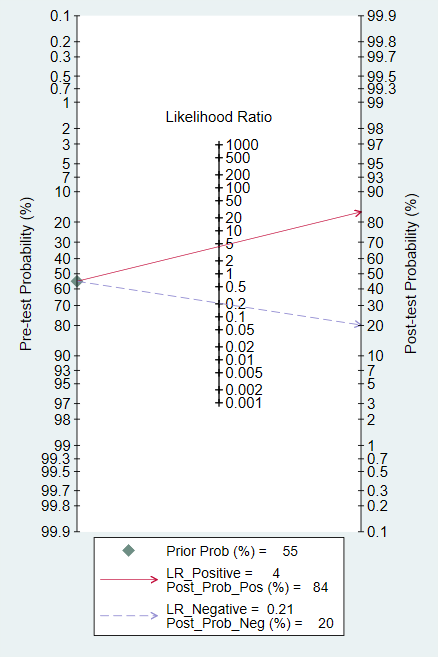


**Figure S45:** The Deek's funnel plot curve of radiomics based on MRI-based models for the diagnosis of pleomorphic adenoma (The training set)


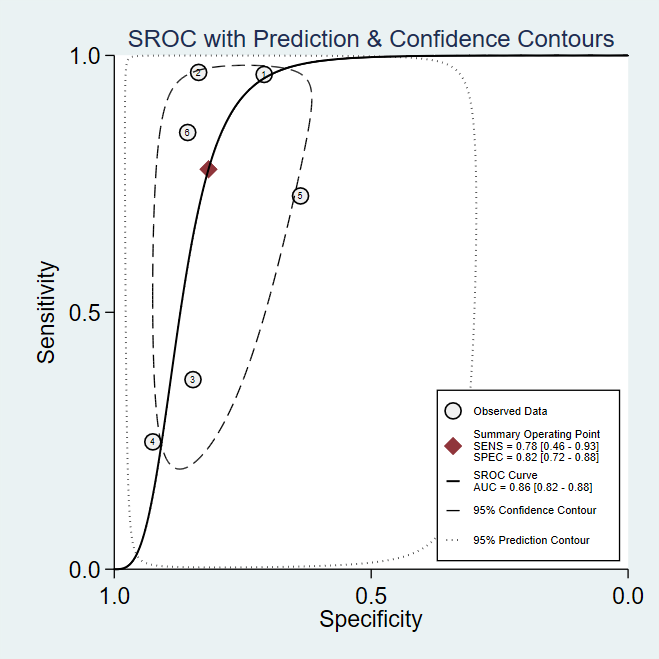


**Figure S46:** The SROC curve based on clinical features for the diagnosis of pleomorphic adenoma (The training set)


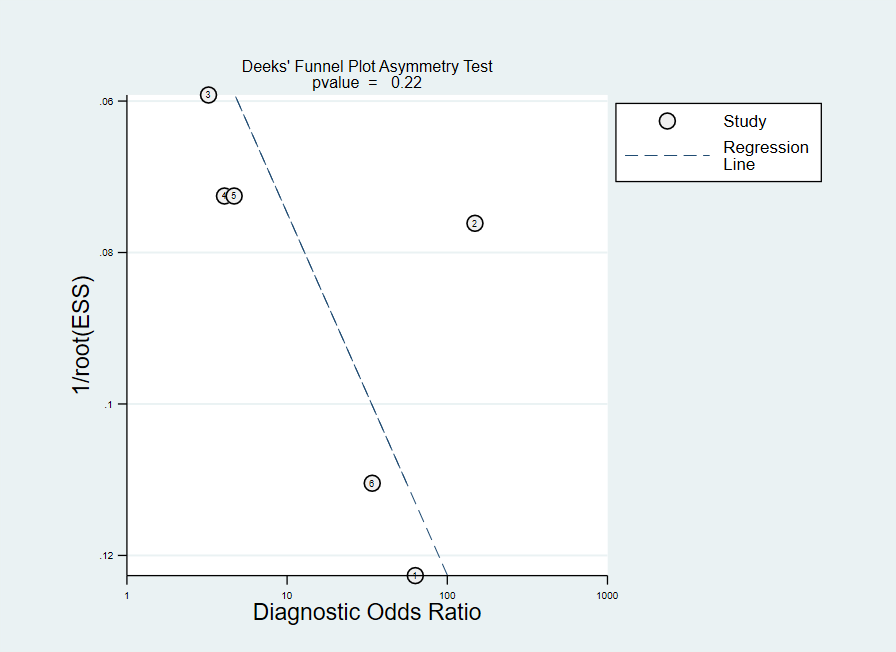


**Figure S47:** The Deek's funnel plot curve based on clinical features for the diagnosis of pleomorphic adenoma (The training set)


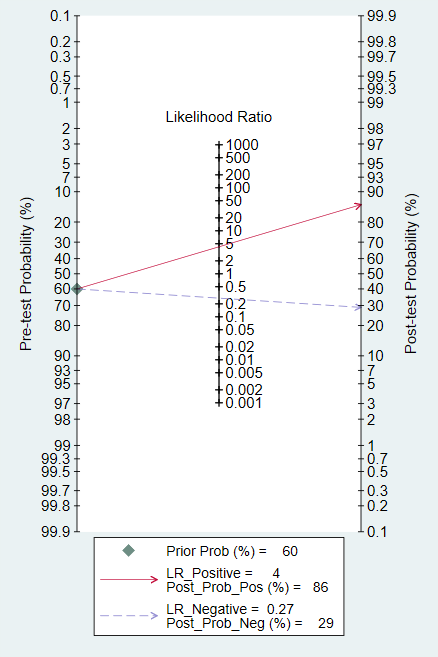


**Figure S48:** The Deek's funnel plot curve based on clinical features for the diagnosis of pleomorphic adenoma (The training set)


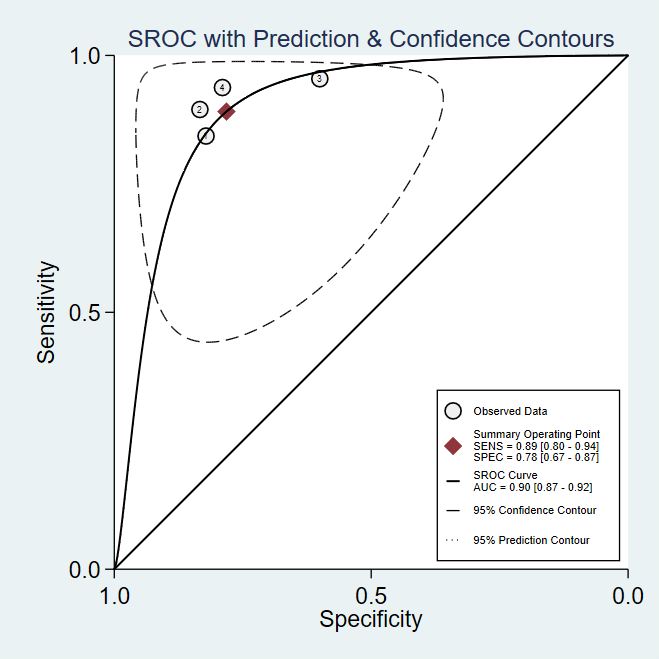


**Figure S49:** The SROC curve based on clinical features for the diagnosis of pleomorphic adenoma (The validation set)


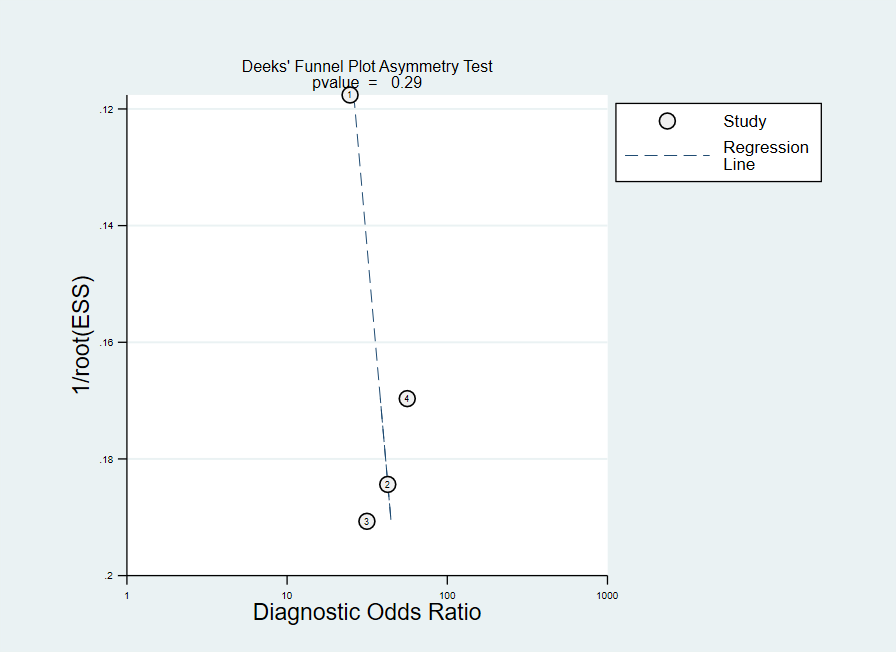


**Figure S50:** The Deek's funnel plot curve based on clinical features for the diagnosis of pleomorphic adenoma (The validation set)


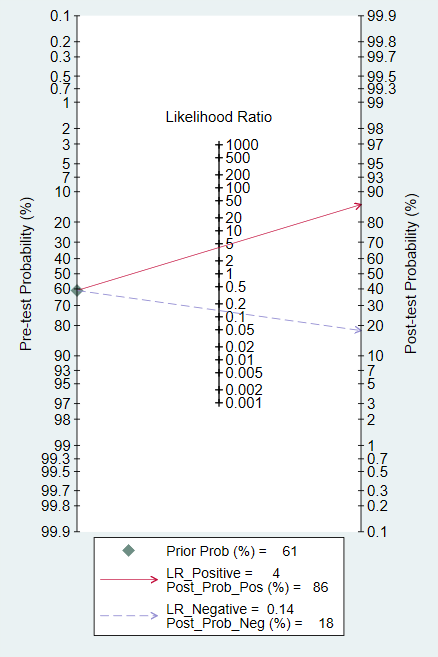


**Figure S51:** The Deek's funnel plot curve based on clinical features for the diagnosis of pleomorphic adenoma (The validation set)


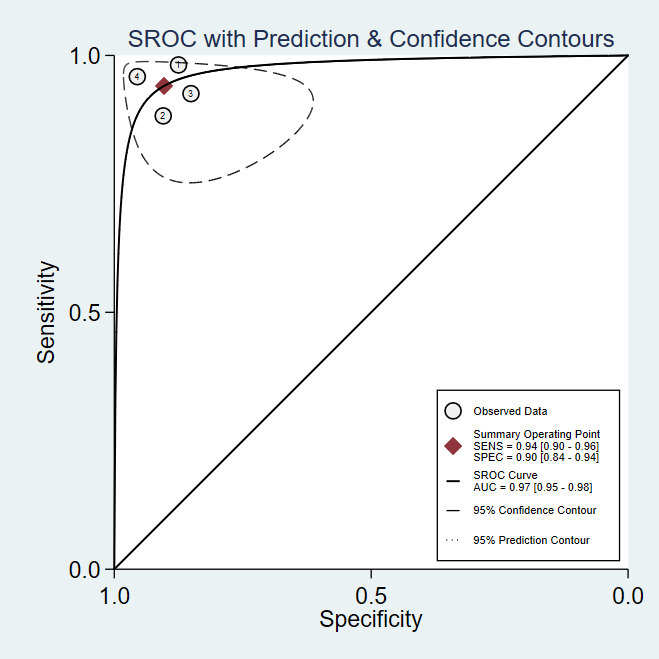


**Figure S52:** The SROC curve based on CT-based models combined with clinical features for the diagnosis of pleomorphic adenoma (The training set)


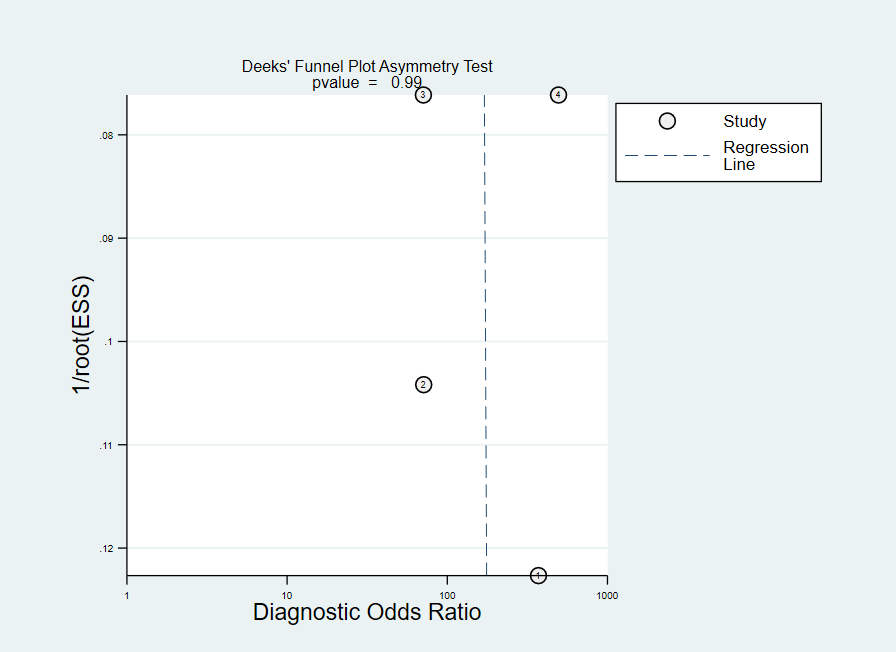


**Figure S53:** The Deek's funnel plot curve based on CT-based models combined with for the diagnosis of pleomorphic adenoma (The training set)


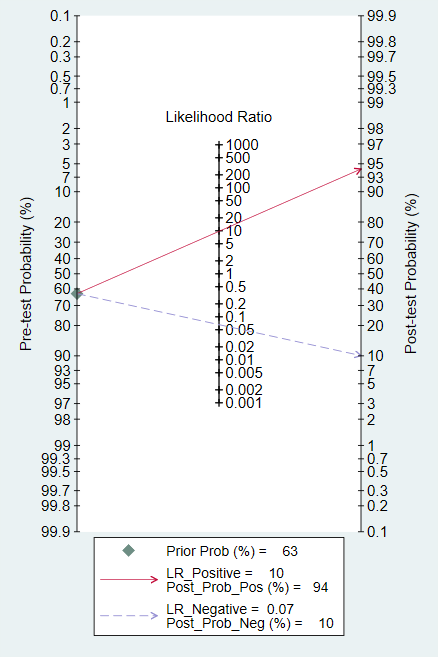


**Figure S54:** The Deek's funnel plot curve based on CT-based models combined with for the diagnosis of pleomorphic adenoma (The training set)
