# Supplementary material for: Performance of radiomics in the differential diagnosis of parotid tumors: a systematic review
Source: Front Oncol. 2024 Jul 25;14:1383323. doi: 10.3389/fonc.2024.1383323 (PMC11306159; doi:10.3389/fonc.2024.1383323)
Supplement: Supplementary file 2 [file DataSheet_2.docx]

**Annex 1: Search strategy**

**Cochrane-30**

Search Name: Parotid tumor and Radiomic

Date Run: 05/05/2023 16:56:11

Comment:

| ID Search Hits |
| --- |
| #1 MeSH descriptor: [Parotid Neoplasms] explode all trees 68 |
| #2 (Parotid Neoplasms):ti,ab,kw OR (Parotid adenoma):ti,ab,kw OR (Parotid Neoplasm):ti,ab,kw OR (Cancer of Parotid):ti,ab,kw OR (Parotid Cancers):ti,ab,kw 382 |
| #3 (Parotid Cancer):ti,ab,kw OR (Cancer of the Parotid):ti,ab,kw OR (parotid gland):ti,ab,kw OR (parotid glands):ti,ab,kw OR (parotid tumors):ti,ab,kw 657 |
| #4 (parotid tumor):ti,ab,kw OR (parotid gland tumor):ti,ab,kw OR (parotid neoplasms):ti,ab,kw OR (parotid tumour):ti,ab,kw OR (parotis tumor):ti,ab,kw 301 |
| #5 (parotis tumour):ti,ab,kw 0 |
| #6 #1 OR #2 OR #3 OR #4 OR #5 672 |
| #7 (Radiomics):ti,ab,kw OR (radiomic):ti,ab,kw OR (radiogenomic):ti,ab,kw OR (radiomics-based):ti,ab,kw OR (radiomic signature):ti,ab,kw 578 |
| #8 (Texture):ti,ab,kw OR (machine learning):ti,ab,kw OR (Transfer Learning):ti,ab,kw OR (Deep learning):ti,ab,kw OR (Ensemble Learning):ti,ab,kw 6802 |
| #9 (artificial intelligence):ti,ab,kw AND (random forest):ti,ab,kw AND (neural network):ti,ab,kw AND (neural networks):ti,ab,kw AND (CNN):ti,ab,kw 1 |
| #10 (Support vector machine):ti,ab,kw OR (SVM):ti,ab,kw OR (Gradient Boosting Machine):ti,ab,kw OR (Nomogram):ti,ab,kw OR (XGBoost):ti,ab,kw 2079 |
| #11 (Adaboost):ti,ab,kw OR (Decision tree):ti,ab,kw OR (ResNet-50):ti,ab,kw OR (ResNet):ti,ab,kw OR (Naive Bayesian):ti,ab,kw 1013 |
| #12 (Multilayer perceptron):ti,ab,kw OR (Bayesian network):ti,ab,kw 330 |
| #13 #7 OR #8 OR #9 OR #10 OR #11 OR #12 9651 |
| #14 #6 AND #13 30 |

**Embase-301**

| No. | Query | Results | Date |
| --- | --- | --- | --- |
| #7 | #3 AND #6 | 301 | ####### |
| #6 | #4 OR #5 | 332173 | ####### |
| #5 | radiomic:ab,ti OR radiogenomic:ab,ti OR 'radiomics based':ab,ti OR 'radiomic signature':ab,ti OR texture:ab,ti OR 'machine learning':ab,ti OR 'transfer learning':ab,ti OR 'deep learning':ab,ti OR 'ensemble learning':ab,ti OR 'artificial intelligence':ab,ti OR 'random forest':ab,ti OR 'neural network':ab,ti OR 'neural networks':ab,ti OR cnn:ab,ti OR 'support vector machine':ab,ti OR svm:ab,ti OR 'gradient boosting machine':ab,ti OR nomogram:ab,ti OR xgboost:ab,ti OR adaboost:ab,ti OR 'decision tree':ab,ti OR 'resnet 50':ab,ti OR resnet:ab,ti OR 'naive bayesian':ab,ti OR 'multilayer perceptron':ab,ti OR 'bayesian network':ab,ti | 330491 | ####### |
| #4 | 'radiomics'/exp | 7579 | ####### |
| #3 | #1 OR #2 | 24373 | ####### |
| #2 | 'parotid gland tumor':ab,ti OR 'parotid adenoma':ab,ti OR 'parotid neoplasm':ab,ti OR 'cancer of parotid':ab,ti OR 'parotid cancers':ab,ti OR 'parotid cancer':ab,ti OR 'cancer of the parotid':ab,ti OR 'parotid gland':ab,ti OR 'parotid glands':ab,ti OR 'parotid tumors':ab,ti OR 'parotid tumor':ab,ti OR 'parotid neoplasms':ab,ti OR 'parotid tumour':ab,ti OR 'parotis tumor':ab,ti OR 'parotis tumour':ab,ti | 20163 | ####### |
| #1 | 'parotid gland tumor'/exp | 10859 | ####### |

**Pubmed-177**

| Search number | Query | Sort By | Filters | Search Details | Results | Time |
| --- | --- | --- | --- | --- | --- | --- |
| 8 | (("Parotid Neoplasms"[Mesh]) OR ((((((((((((((((Parotid Neoplasms[Title/Abstract]) OR (Parotid adenoma[Title/Abstract])) OR (Parotid Neoplasm[Title/Abstract])) OR (Cancer of Parotid[Title/Abstract])) OR (Parotid Cancers[Title/Abstract])) OR (Parotid Cancer[Title/Abstract])) OR (Cancer of the Parotid[Title/Abstract])) OR (parotid gland[Title/Abstract])) OR (parotid glands[Title/Abstract])) OR (parotid tumors[Title/Abstract])) OR (parotid tumor[Title/Abstract])) OR (parotid gland tumor[Title/Abstract])) OR (parotid neoplasms[Title/Abstract])) OR (parotid tumour[Title/Abstract])) OR (parotis tumor[Title/Abstract])) OR (parotis tumour[Title/Abstract]))) AND (((((((((((((((((((((((((((Radiomics[Title/Abstract]) OR (radiomic[Title/Abstract])) OR (radiogenomic[Title/Abstract])) OR (radiomics-based[Title/Abstract])) OR (radiomic signature[Title/Abstract])) OR (Texture[Title/Abstract])) OR (machine learning[Title/Abstract])) OR (Transfer Learning[Title/Abstract])) OR (Deep learning[Title/Abstract])) OR (Ensemble Learning[Title/Abstract])) OR (artificial intelligence[Title/Abstract])) OR (random forest[Title/Abstract])) OR (neural network[Title/Abstract])) OR (neural networks[Title/Abstract])) OR (CNN[Title/Abstract])) OR (Support vector machine[Title/Abstract])) OR (SVM[Title/Abstract])) OR (Gradient Boosting Machine[Title/Abstract])) OR (Nomogram[Title/Abstract])) OR (XGBoost[Title/Abstract])) OR (Adaboost[Title/Abstract])) OR (Decision tree[Title/Abstract])) OR (ResNet-50[Title/Abstract])) OR (ResNet[Title/Abstract])) OR (Naive Bayesian[Title/Abstract])) OR (Multilayer perceptron[Title/Abstract])) OR (Bayesian network[Title/Abstract])) | | | ("Parotid Neoplasms"[MeSH Terms] OR ("Parotid Neoplasms"[Title/Abstract] OR "parotid adenoma"[Title/Abstract] OR "parotid neoplasm"[Title/Abstract] OR "cancer of parotid"[Title/Abstract] OR "parotid cancers"[Title/Abstract] OR "parotid cancer"[Title/Abstract] OR "cancer of the parotid"[Title/Abstract] OR "parotid gland"[Title/Abstract] OR "parotid glands"[Title/Abstract] OR "parotid tumors"[Title/Abstract] OR "parotid tumor"[Title/Abstract] OR "parotid gland tumor"[Title/Abstract] OR "Parotid Neoplasms"[Title/Abstract] OR "parotid tumour"[Title/Abstract] OR ("parotis"[All Fields] AND "tumor"[Title/Abstract]) OR ("parotis"[All Fields] AND "tumour"[Title/Abstract]))) AND ("Radiomics"[Title/Abstract] OR "radiomic"[Title/Abstract] OR "radiogenomic"[Title/Abstract] OR "radiomics-based"[Title/Abstract] OR "radiomic signature"[Title/Abstract] OR "Texture"[Title/Abstract] OR "machine learning"[Title/Abstract] OR "transfer learning"[Title/Abstract] OR "deep learning"[Title/Abstract] OR "ensemble learning"[Title/Abstract] OR "artificial intelligence"[Title/Abstract] OR "random forest"[Title/Abstract] OR "neural network"[Title/Abstract] OR "neural networks"[Title/Abstract] OR "CNN"[Title/Abstract] OR "support vector machine"[Title/Abstract] OR "SVM"[Title/Abstract] OR "gradient boosting machine"[Title/Abstract] OR "Nomogram"[Title/Abstract] OR "XGBoost"[Title/Abstract] OR "Adaboost"[Title/Abstract] OR "decision tree"[Title/Abstract] OR "ResNet-50"[Title/Abstract] OR "ResNet"[Title/Abstract] OR "naive bayesian"[Title/Abstract] OR "multilayer perceptron"[Title/Abstract] OR "bayesian network"[Title/Abstract]) | 177 | 10:19:58 |
| 7 | ((((((((((((((((((((((((((Radiomics[Title/Abstract]) OR (radiomic[Title/Abstract])) OR (radiogenomic[Title/Abstract])) OR (radiomics-based[Title/Abstract])) OR (radiomic signature[Title/Abstract])) OR (Texture[Title/Abstract])) OR (machine learning[Title/Abstract])) OR (Transfer Learning[Title/Abstract])) OR (Deep learning[Title/Abstract])) OR (Ensemble Learning[Title/Abstract])) OR (artificial intelligence[Title/Abstract])) OR (random forest[Title/Abstract])) OR (neural network[Title/Abstract])) OR (neural networks[Title/Abstract])) OR (CNN[Title/Abstract])) OR (Support vector machine[Title/Abstract])) OR (SVM[Title/Abstract])) OR (Gradient Boosting Machine[Title/Abstract])) OR (Nomogram[Title/Abstract])) OR (XGBoost[Title/Abstract])) OR (Adaboost[Title/Abstract])) OR (Decision tree[Title/Abstract])) OR (ResNet-50[Title/Abstract])) OR (ResNet[Title/Abstract])) OR (Naive Bayesian[Title/Abstract])) OR (Multilayer perceptron[Title/Abstract])) OR (Bayesian network[Title/Abstract]) | | | "Radiomics"[Title/Abstract] OR "radiomic"[Title/Abstract] OR "radiogenomic"[Title/Abstract] OR "radiomics-based"[Title/Abstract] OR "radiomic signature"[Title/Abstract] OR "Texture"[Title/Abstract] OR "machine learning"[Title/Abstract] OR "transfer learning"[Title/Abstract] OR "deep learning"[Title/Abstract] OR "ensemble learning"[Title/Abstract] OR "artificial intelligence"[Title/Abstract] OR "random forest"[Title/Abstract] OR "neural network"[Title/Abstract] OR "neural networks"[Title/Abstract] OR "CNN"[Title/Abstract] OR "support vector machine"[Title/Abstract] OR "SVM"[Title/Abstract] OR "gradient boosting machine"[Title/Abstract] OR "Nomogram"[Title/Abstract] OR "XGBoost"[Title/Abstract] OR "Adaboost"[Title/Abstract] OR "decision tree"[Title/Abstract] OR "ResNet-50"[Title/Abstract] OR "ResNet"[Title/Abstract] OR "naive bayesian"[Title/Abstract] OR "multilayer perceptron"[Title/Abstract] OR "bayesian network"[Title/Abstract] | 290,523 | 10:19:20 |
| 6 | ("Parotid Neoplasms"[Mesh]) OR ((((((((((((((((Parotid Neoplasms[Title/Abstract]) OR (Parotid adenoma[Title/Abstract])) OR (Parotid Neoplasm[Title/Abstract])) OR (Cancer of Parotid[Title/Abstract])) OR (Parotid Cancers[Title/Abstract])) OR (Parotid Cancer[Title/Abstract])) OR (Cancer of the Parotid[Title/Abstract])) OR (parotid gland[Title/Abstract])) OR (parotid glands[Title/Abstract])) OR (parotid tumors[Title/Abstract])) OR (parotid tumor[Title/Abstract])) OR (parotid gland tumor[Title/Abstract])) OR (parotid neoplasms[Title/Abstract])) OR (parotid tumour[Title/Abstract])) OR (parotis tumor[Title/Abstract])) OR (parotis tumour[Title/Abstract])) | | | "Parotid Neoplasms"[MeSH Terms] OR ("Parotid Neoplasms"[Title/Abstract] OR "parotid adenoma"[Title/Abstract] OR "parotid neoplasm"[Title/Abstract] OR "cancer of parotid"[Title/Abstract] OR "parotid cancers"[Title/Abstract] OR "parotid cancer"[Title/Abstract] OR "cancer of the parotid"[Title/Abstract] OR "parotid gland"[Title/Abstract] OR "parotid glands"[Title/Abstract] OR "parotid tumors"[Title/Abstract] OR "parotid tumor"[Title/Abstract] OR "parotid gland tumor"[Title/Abstract] OR "Parotid Neoplasms"[Title/Abstract] OR "parotid tumour"[Title/Abstract] OR ("parotis"[All Fields] AND "tumor"[Title/Abstract]) OR ("parotis"[All Fields] AND "tumour"[Title/Abstract])) | 20,567 | 10:15:09 |
| 5 | (((((((((((((((Parotid Neoplasms[Title/Abstract]) OR (Parotid adenoma[Title/Abstract])) OR (Parotid Neoplasm[Title/Abstract])) OR (Cancer of Parotid[Title/Abstract])) OR (Parotid Cancers[Title/Abstract])) OR (Parotid Cancer[Title/Abstract])) OR (Cancer of the Parotid[Title/Abstract])) OR (parotid gland[Title/Abstract])) OR (parotid glands[Title/Abstract])) OR (parotid tumors[Title/Abstract])) OR (parotid tumor[Title/Abstract])) OR (parotid gland tumor[Title/Abstract])) OR (parotid neoplasms[Title/Abstract])) OR (parotid tumour[Title/Abstract])) OR (parotis tumor[Title/Abstract])) OR (parotis tumour[Title/Abstract]) | | | "parotid neoplasms"[Title/Abstract] OR "parotid adenoma"[Title/Abstract] OR "parotid neoplasm"[Title/Abstract] OR "cancer of parotid"[Title/Abstract] OR "parotid cancers"[Title/Abstract] OR "parotid cancer"[Title/Abstract] OR "cancer of the parotid"[Title/Abstract] OR "parotid gland"[Title/Abstract] OR "parotid glands"[Title/Abstract] OR "parotid tumors"[Title/Abstract] OR "parotid tumor"[Title/Abstract] OR "parotid gland tumor"[Title/Abstract] OR "parotid neoplasms"[Title/Abstract] OR "parotid tumour"[Title/Abstract] OR ("parotis"[All Fields] AND "tumor"[Title/Abstract]) OR ("parotis"[All Fields] AND "tumour"[Title/Abstract]) | 17,117 | 10:12:29 |
| 4 | "Parotid Neoplasms"[Mesh] | Most Recent | | "Parotid Neoplasms"[MeSH Terms] | 9,436 | 10:09:26 |
| 3 | Radiomics | | Meta-Analysis | ("radiomic"[All Fields] OR "radiomics"[All Fields]) AND (meta-analysis[Filter]) | 43 | 9:55:08 |
| 2 | Radiomics | |  | "radiomic"[All Fields] OR "radiomics"[All Fields] | 8,332 | 9:55:04 |
| 1 | Parotid Neoplasms | |  | "parotid neoplasms"[MeSH Terms] OR ("parotid"[All Fields] AND "neoplasms"[All Fields]) OR "parotid neoplasms"[All Fields] | 13,976 | 9:54:45 |

**WoS-300**

| limits authority | # | Search Details | Databases | Results | Date |
| --- | --- | --- | --- | --- | --- |
| - WOS.IC: 1993 to 2023 - WOS.CCR: 1985 to 2023 - WOS.SCI: 1975 to 2023 - WOS.AHCI: 1975 to 2023 - WOS.BHCI: 2005 to 2023 - WOS.BSCI: 2005 to 2023 - WOS.ESCI: 2018 to 2023 - WOS.ISTP: 1990 to 2023 - WOS.SSCI: 1965 to 2023 - WOS.ISSHP: 1990 to 2023 | 1 | Parotid Neoplasms (Topic) OR Parotid adenoma (Topic) OR Parotid Neoplasm (Topic) OR Cancer of Parotid (Topic) OR Parotid Cancers (Topic) OR Parotid Cancer (Topic) OR Cancer of the Parotid (Topic) OR parotid gland (Topic) OR parotid glands (Topic) OR parotid tumors (Topic) OR parotid tumor (Topic) OR parotid gland tumor (Topic) OR parotid neoplasms (Topic) OR parotid tumour (Topic) OR parotis tumor (Topic) OR parotis tumour (Topic) | Web of Science | 18593 | Fri May 05 2023 23:02:28 GMT+0800 |
| - WOS.IC: 1993 to 2023 - WOS.CCR: 1985 to 2023 - WOS.SCI: 1975 to 2023 - WOS.AHCI: 1975 to 2023 - WOS.BHCI: 2005 to 2023 - WOS.BSCI: 2005 to 2023 - WOS.ESCI: 2018 to 2023 - WOS.ISTP: 1990 to 2023 - WOS.SSCI: 1965 to 2023 - WOS.ISSHP: 1990 to 2023 | 2 | Radiomics (Topic) OR radiomic (Topic) OR radiogenomic (Topic) OR radiomics-based (Topic) OR radiomic signature (Topic) OR Texture (Topic) OR machine learning (Topic) OR Transfer Learning (Topic) OR Deep learning (Topic) OR Ensemble Learning (Topic) OR artificial intelligence (Topic) OR random forest (Topic) OR neural network (Topic) OR neural networks (Topic) OR CNN (Topic) OR Support vector machine (Topic) OR SVM (Topic) OR Gradient Boosting Machine (Topic) OR Nomogram (Topic) OR XGBoost (Topic) OR Adaboost (Topic) OR Decision tree (Topic) OR ResNet-50 (Topic) OR ResNet (Topic) OR Naive Bayesian (Topic) OR Multilayer perceptron (Topic) OR Bayesian network (Topic) | Web of Science | 1592830 | Fri May 05 2023 23:05:42 GMT+0800 |
| - WOS.IC: 1993 to 2023 - WOS.CCR: 1985 to 2023 - WOS.SCI: 1975 to 2023 - WOS.AHCI: 1975 to 2023 - WOS.BHCI: 2005 to 2023 - WOS.BSCI: 2005 to 2023 - WOS.ESCI: 2018 to 2023 - WOS.ISTP: 1990 to 2023 - WOS.SSCI: 1965 to 2023 - WOS.ISSHP: 1990 to 2023 | 3 | #2 AND #1 | Web of Science | 300 | Fri May 05 2023 23:06:17 GMT+0800 |

**Annex 2: The radiomics quality score: RQS**

| **Criteria** | | **Points** |
| --- | --- | --- |
| 1 | Image protocol quality - well-documented image protocols (for example, contrast, slice thickness, energy, etc.) and/or usage of public image protocols allow reproducibility/replicability | + 1 (if protocols are well-documented) + 1 (if public protocol is used) |
| 2 | Multiple segmentations - possible actions are: segmentation by different physicians/algorithms/software, perturbing segmentations by (random) noise, segmentation at different breathing cycles. Analyse feature robustness to segmentation variabilities | + 1 |
| 3 | Phantom study on all scanners - detect inter-scanner differences and vendor-dependent features. Analyse feature robustness to these sources of variability | + 1 |
| 4 | Imaging at multiple time points - collect images of individuals at additional time points. Analyse feature robustness to temporal variabilities (for example, organ movement, organ expansion/ shrinkage) | + 1 |
| 5 | Feature reduction or adjustment for multiple testing - decreases the risk of overfitting. Overfitting is inevitable if the number of features exceeds the number of samples. Consider feature robustness when selecting features | - 3 (if neither measure is implemented) + 3 (if either measure is implemented) |
| 6 | Multivariable analysis with non radiomics features (for example, EGFR mutation) - is expected to provide a more holistic model. Permits correlating/inferencing between radiomics and non radiomics features | + 1 |
| 7 | Detect and discuss biological correlates - demonstration of phenotypic differences (possibly associated with underlying gene–protein expression patterns) deepens understanding of radiomics and biology | + 1 |
| 8 | Cut-off analyses - determine risk groups by either the median, a previously published cut-off or report a continuous risk variable. Reduces the risk of reporting overly optimistic results | + 1 |
| 9 | Discrimination statistics - report discrimination statistics (for example,  C-statistic, ROC curve, AUC) and their statistical significance (for example, p-values, confidence intervals). One can also apply  resampling method (for example, bootstrapping, cross-validation) | + 1 (if a discrimination statistic and its statistical significance are reported) + 1 (if a resampling method technique is also applied) |
| 10 | Calibration statistics - report calibration statistics (for example, Calibration-in-the-large/slope, calibration plots) and their statistical significance (for example, *P*-values, confidence intervals). One  can also apply resampling method (for example, bootstrapping, cross-validation) | + 1 (if a calibration statistic and its statistical significance are reported) + 1 (if a resampling method technique is also applied) |
| 11 | Prospective study registered in a trial database - provides the highest level of evidence supporting the clinical validity and usefulness of the radiomics biomarker | + 7 (for prospective validation of a radiomics signature in an appropriate trial) |
| 12 | Validation - the validation is performed without retraining and without adaptation of the cut-off value, provides crucial information with regard to credible clinical performance | - 5 (if validation is missing) + 2 (if validation is based on a dataset from the same institute) + 3 (if validation is based on a dataset from another institute) + 4 (if validation is based on two datasets from two distinct institutes) + 4 (if the study validates a previously published signature) + 5 (if validation is based on three or more datasets from distinct institutes)  *Datasets should be of comparable size and should have at least 10 events per model feature |
| 13 | Comparison to ‘gold standard’ - assess the extent to which the model agrees with/is superior to the current ‘gold standard’ method (for example, TNM-staging for survival prediction). This comparison shows the added value of radiomics | + 2 |
| 14 | Potential clinical utility - report on the current and potential application of the model in a clinical setting (for example, decision curve analysis). | + 2 |
| 15 | Cost-effectiveness analysis - report on the cost-effectiveness of the clinical application (for example, QALYs generated) | + 1 |
| 16 | Open science and data - make code and data publicly available. Open science facilitates knowledge transfer and reproducibility of the study | + 1 (if scans are open source) + 1 (if region of interest segmentations are open source) + 1 (if code is open source)  + 1 (if radiomics features are calculated on a set of representative ROIs and the calculated features and representative ROIs are open source) |
| Total points (36 = 100%) | | |

**Annex 3:** The mean RQS score for the included studies.

| No | Lead author | Publication time | v1 | v2 | v3 | v4 | v5 | v6 | v7 | v8 | v9 | v10 | v11 | v12 | v13 | v14 | v15 | v16 | Total | Proportion |
| --- | --- | --- | --- | --- | --- | --- | --- | --- | --- | --- | --- | --- | --- | --- | --- | --- | --- | --- | --- | --- |
| 33 | Xu Liu | 2023 | 1 |  |  |  |  |  |  |  | 2 |  |  | 2 |  |  |  |  | 5 | 13.89 |
| 34 | Yunlin Zheng | 2022 | 1 | 1 |  |  |  | 1 |  |  | 2 |  |  | 2 |  | 2 |  | 1 | 10 | 27.78 |
| 35 | Qiang Yu | 2023 | 1 | 1 |  |  |  | 1 |  |  | 1 |  |  | 3 |  |  |  | 1 | 8 | 22.22 |
| 36 | Michela Gabelloni | 2020 | 1 |  |  |  | -3 |  |  |  | 1 |  |  | 2 |  |  |  |  | 1 | 2.78 |
| 37 | Hidetoshi Matsuo | 2020 | 1 | 1 |  |  | -3 |  |  |  | 1 |  |  | 2 |  | 2 |  |  | 4 | 11.11 |
| 38 | Eiman Al Ajmi | 2018 | 1 | 1 |  |  |  |  |  |  | 2 |  |  | 2 |  |  |  |  | 6 | 16.67 |
| 39 | Henry J. Areiza-Laverdea | 2020 | 1 |  |  |  |  |  |  |  | 2 |  |  | 2 |  |  |  |  | 5 | 13.89 |
| 40 | Baohong Wen | 2022 | 1 | 1 |  |  |  |  |  |  | 2 |  |  | 2 |  |  |  | 1 | 7 | 19.44 |
| 41 | Yi-Ju Chang | 2020 | 1 |  |  |  |  |  |  |  | 2 |  |  | 2 |  |  |  | 1 | 6 | 16.67 |
| 42 | Yun-lin Zheng | 2022 | 1 | 1 |  |  |  |  |  |  | 2 |  |  | 2 |  |  |  | 1 | 7 | 19.44 |
| 43 | Xianwu Xia | 2021 | 1 | 1 |  |  | -3 |  |  |  | 1 |  |  | 2 |  |  |  | 1 | 3 | 8.33 |
| 44 | Xue-Meng Shen | 2022 | 1 | 1 |  |  | -3 |  |  |  | 1 |  |  | 2 |  | 2 |  | 1 | 5 | 13.89 |
| 45 | Ying-mei Zheng | 2021 | 1 | 1 |  |  |  | 1 |  | 1 | 2 | 2 |  | 3 |  | 2 |  |  | 13 | 36.11 |
| 46 | Menglong Zheng | 2022 | 1 | 1 |  |  |  | 1 |  | 1 | 1 | 2 |  | 2 |  | 2 |  |  | 11 | 30.56 |
| 47 | Yushuai Yuan | 2021 | 1 | 1 |  |  |  |  |  |  | 2 |  |  | 2 |  |  |  |  | 6 | 16.67 |
| 48 | Jinbo Qi | 2022 | 1 | 1 |  |  |  | 1 |  | 1 | 2 | 2 |  | 2 |  | 2 |  | 1 | 13 | 36.11 |
| 49 | Baomin Feng, | 2023 | 1 | 1 |  |  |  | 1 |  | 1 | 1 | 2 |  | 3 |  | 2 |  | 1 | 13 | 36.11 |
| 50 | Chun-Jung Juan | 2020 | 1 |  |  |  | -3 |  |  |  | 1 |  |  | 2 |  |  |  | 1 | 2 | 5.56 |
| 51 | Dan Zhang | 2020 | 1 | 1 |  |  | -3 | 1 |  |  | 1 |  |  | -5 |  |  |  |  | -4 | -11.11 |
| 52 | Zhiying He | 2022 | 1 | 1 |  |  |  | 1 |  |  | 2 |  |  | 2 |  |  |  | 1 | 8 | 22.22 |
| 53 | Yuebo Liu | 2021 | 1 | 1 |  |  |  | 1 |  |  | 2 |  |  | 2 |  | 2 |  | 1 | 10 | 27.78 |
| 54 | Ying-mei Zheng | 2020 | 1 | 1 |  |  |  | 1 |  |  | 2 | 2 |  | 3 |  | 2 |  | 1 | 13 | 36.11 |
| 55 | Francesca Piludu | 2021 | 1 | 1 |  |  |  |  |  | 1 | 2 |  |  | 3 |  |  |  | 1 | 9 | 25.00 |
| 56 | Qiang Yu | 2022 | 1 | 1 |  |  |  | 1 |  |  | 1 | 2 |  | 2 |  | 2 |  | 1 | 11 | 30.56 |
| 57 | Anahita Fathi Kazerooni | 2022 | 1 |  |  |  |  |  |  |  | 1 |  |  | 2 |  |  |  |  | 4 | 11.11 |
| 58 | Yuebo Liu | 2021 | 1 |  |  |  |  | 1 |  | 1 | 2 |  |  | 2 |  |  |  | 1 | 8 | 22.22 |
| 59 | HONGBIN ZHANG | 2021 | 1 |  |  |  |  |  |  |  | 2 |  |  | 2 |  |  |  | 0 | 5 | 13.89 |
| 60 | Yaoqin Wang | 2022 |  | 1 |  |  | -3 |  |  |  | 1 |  |  | 2 |  | 2 |  | 1 | 4 | 11.11 |
| 61 | Yuyun Xu | 2021 | 1 | 1 |  |  | -3 | 1 |  | 1 | 1 | 2 |  | 3 |  | 2 |  | 1 | 10 | 27.78 |
| 62 | Lorenzo Faggioni | 2022 |  |  |  |  | -3 |  |  |  | 1 |  |  | -5 |  |  |  |  | -7 | -19.44 |
| 63 | Ziyang Hu | 2022 | 1 | 1 |  |  |  |  |  |  | 2 |  |  | 2 |  | 2 |  | 1 | 9 | 25.00 |
| 64 | Zhenbin Hu | 2023 | 1 | 1 |  |  |  | 1 |  | 1 | 2 | 2 |  | 2 |  | 2 |  | 1 | 13 | 36.11 |
| 71 | Yang Lu | 2023 | 1 | 1 |  |  |  |  |  |  | 2 |  |  | 2 |  |  |  |  | 6 | 16.67 |
| 72 | Delia Doris Muntean | 2023 | 1 |  |  |  |  | 1 |  |  | 2 |  |  | 2 |  |  |  | 1 | 7 | 19.44 |
| 73 | Xue-Meng Shen | 2023 | 1 | 1 |  |  | -3 |  |  |  | 1 |  |  | 2 |  | 2 |  | 1 | 5 | 13.89 |
| 74 | Fangfang Chen | 2023 | 1 | 1 |  |  |  |  |  | 1 | 2 | 2 |  | 2 |  |  |  | 1 | 10 | 27.78 |
